# Supplementary material for: Th1 polarization in Bordetella pertussis vaccine responses is maintained through a positive feedback loop
Source: bioRxiv. 2024 Oct 17:2024.08.05.606623. Originally published 2024 Aug 7. Preprint. [Version 2] doi: 10.1101/2024.08.05.606623 (PMC11326151; doi:10.1101/2024.08.05.606623)
Supplement: Supplement 1 [file media-1.pdf]

Supplementary Table 1. Study population characteristics. Shapiro-Wilk and Mann-Whitney tests were performed to test differences in age at booster between the aP and wP groups. A Chi-square test was used to check if biological sex differed per group.

	**aP (n=31)**	**wP (n=30)**	***P*-value**
**Age at booster, mean years ± SD**	21.1 ± 2.2	31.0 ± 7.2	***P*<0.001**
**Sex, n male (%)**	10 (32.3%)	10 (33.3%)	*P*=0.929

Supplementary Table 2. Performed assays per donor.

Doras_ID	Subject_ID	Group	AIM	Fluorospot	Plasma antibodies	Plasma cytokines	RNAseq
6797	0	wP	YES	YES	YES	YES	NO
1686	2	wP	YES	YES	YES	YES	NO
2631	8	wP	YES	YES	YES	YES	NO
2726	26	wP	NO	NO	NO	YES	NO
2903	45	aP	NO	NO	NO	YES	NO
3664	61	wP	YES	YES	YES	YES	YES
3802	62	wP	YES	YES	YES	YES	YES
3803	63	wP	YES	YES	YES	YES	YES
3804	64	wP	YES	YES	YES	YES	YES
3806	65	wP	YES	NO	YES	YES	YES
3808	66	wP	YES	YES	YES	YES	YES
3943	67	wP	YES	YES	YES	YES	YES
3944	68	wP	YES	YES	YES	YES	YES
3945	69	wP	YES	YES	YES	YES	YES
3946	70	aP	YES	YES	YES	YES	YES
3947	71	aP	YES	YES	YES	YES	YES
3984	72	wP	YES	YES	YES	YES	YES
3985	73	wP	YES	YES	YES	YES	YES
3986	74	wP	YES	YES	YES	YES	YES
3987	75	aP	YES	YES	YES	YES	YES
3989	76	aP	YES	YES	YES	YES	YES
4016	77	wP	YES	YES	YES	YES	YES
4017	78	wP	YES	YES	YES	YES	YES
4018	79	wP	YES	YES	YES	YES	YES
4019	80	wP	YES	NO	YES	YES	YES
4020	81	wP	YES	YES	YES	YES	YES
4021	82	aP	YES	YES	NO	YES	YES

4054	83	aP	YES	NO	YES	YES	YES
4055	84	aP	YES	YES	YES	YES	YES
4089	85	aP	YES	NO	YES	YES	YES
4090	86	aP	YES	YES	YES	YES	YES
4091	87	aP	NO	NO	YES	YES	YES
4092	88	aP	NO	NO	NO	YES	YES
4138	89	aP	YES	YES	YES	YES	YES
4139	90	aP	YES	YES	YES	YES	YES
4140	91	aP	YES	YES	YES	YES	YES
4162	92	aP	YES	YES	YES	YES	YES
4163	93	aP	YES	YES	YES	YES	YES
4164	94	aP	YES	YES	YES	YES	YES
4165	95	aP	YES	YES	YES	YES	YES
4166	96	aP	YES	YES	YES	YES	YES
1788	97	wP	YES	YES	YES	YES	YES
4654	98	wP	YES	YES	YES	YES	YES
4656	99	aP	YES	YES	YES	YES	YES
4657	100	aP	YES	NO	YES	YES	YES
6096	101	aP	YES	YES	YES	YES	YES
6097	102	aP	YES	YES	YES	YES	YES
6275	103	wP	YES	YES	YES	YES	YES
6279	104	wP	YES	YES	YES	YES	YES
6280	105	wP	YES	NO	YES	YES	YES
6281	106	aP	YES	YES	YES	YES	YES
6308	107	aP	YES	YES	YES	YES	YES
6385	108	wP	YES	YES	YES	YES	YES
6388	109	wP	YES	YES	YES	YES	YES
6389	110	aP	YES	YES	YES	YES	YES
6482	111	wP	YES	YES	YES	YES	YES

6483	112	aP	YES	YES	YES	YES	YES
6485	114	wP	YES	YES	YES	YES	YES
6495	115	aP	YES	YES	YES	YES	YES
6679	117	aP	YES	NO	YES	YES	YES
6787	118	aP	YES	YES	YES	YES	YES

Supplementary Table 3. Spearman correlation statistics between gene expression changes (post/pre-b) and T cell polarization (IFN-γ/IL-5 SFC) pre and 28 days post-booster. Significant correlates are shown (P<0.05).

Parameter 1: Th1 polarization	Parameter 2: Gene (Symbol_ENSEMBL ID)	GEX days	r	*P*	n
IFN-γ/IL-5 SFC day 28	GM2A_ENSG00000196743	day1/pre-b	0.515	0.0003	44
IFN-γ/IL-5 SFC day 28	LAPTM5_ENSG00000162511	day1/pre-b	0.512	0.0004	44
IFN-γ/IL-5 SFC day 28	TAPBP_ENSG00000231925	day1/pre-b	0.508	0.0004	44
IFN-γ/IL-5 SFC day 28	MARS1_ENSG00000166986	day1/pre-b	0.506	0.0005	44
IFN-γ/IL-5 SFC pre-b	GBP5_ENSG00000154451	day1/pre-b	0.482	0.0006	47
IFN-γ/IL-5 SFC pre-b	GBP2_ENSG00000162645	day1/pre-b	0.478	0.0007	47
IFN-γ/IL-5 SFC day 28	HLA.DPB1_ENSG00000223865	day1/pre-b	0.488	0.0008	44
IFN-γ/IL-5 SFC pre-b	STAT1_ENSG00000115415	day1/pre-b	0.469	0.0009	47
IFN-γ/IL-5 SFC day 28	X_ENSG00000279861	day1/pre-b	0.483	0.0009	44
IFN-γ/IL-5 SFC day 28	C2_ENSG00000166278	day1/pre-b	0.482	0.0009	44
IFN-γ/IL-5 SFC day 28	NCSTN_ENSG00000162736	day1/pre-b	0.48	0.001	44
IFN-γ/IL-5 SFC day 28	P2RX7_ENSG00000089041	day1/pre-b	0.479	0.001	44
IFN-γ/IL-5 SFC day 28	SUSD6_ENSG00000100647	day1/pre-b	0.478	0.001	44
IFN-γ/IL-5 SFC pre-b	CASP7_ENSG00000165806	day1/pre-b	0.462	0.0011	47
IFN-γ/IL-5 SFC day 28	ATF5_ENSG00000169136	day1/pre-b	0.474	0.0012	44
IFN-γ/IL-5 SFC day 28	ADGRE5_ENSG00000123146	day1/pre-b	0.47	0.0013	44
IFN-γ/IL-5 SFC day 28	APOL4_ENSG00000100336	day1/pre-b	0.47	0.0013	44
IFN-γ/IL-5 SFC day 28	HLA.DMA_ENSG00000204257	day1/pre-b	0.469	0.0013	44
IFN-γ/IL-5 SFC day 28	KIF11_ENSG00000138160	day1/pre-b	0.467	0.0014	44
IFN-γ/IL-5 SFC day 28	USB1_ENSG00000103005	day1/pre-b	0.466	0.0014	44
IFN-γ/IL-5 SFC day 28	NA_ENSG00000234290	day1/pre-b	0.465	0.0015	44
IFN-γ/IL-5 SFC day 28	X_ENSG00000254649	day1/pre-b	0.464	0.0015	44
IFN-γ/IL-5 SFC day 28	TPD52L2_ENSG00000101150	day1/pre-b	0.463	0.0015	44
IFN-γ/IL-5 SFC day 28	RAB8A_ENSG00000167461	day1/pre-b	0.461	0.0016	44
IFN-γ/IL-5 SFC day 28	EFTUD2_ENSG00000108883	day1/pre-b	0.461	0.0017	44
IFN-γ/IL-5 SFC pre-b	WARS1_ENSG00000140105	day1/pre-b	0.445	0.0017	47
IFN-γ/IL-5 SFC day 28	MTCO3P11_ENSG00000237711	day1/pre-b	0.459	0.0017	44
IFN-γ/IL-5 SFC day 28	MAPRE1_ENSG00000101367	day1/pre-b	0.458	0.0017	44
IFN-γ/IL-5 SFC day 28	SEC13_ENSG00000157020	day1/pre-b	0.458	0.0018	44
IFN-γ/IL-5 SFC day 28	MSN_ENSG00000147065	day1/pre-b	0.457	0.0018	44
IFN-γ/IL-5 SFC pre-b	APOL4_ENSG00000100336	day1/pre-b	0.441	0.0019	47
IFN-γ/IL-5 SFC day 28	GNS_ENSG00000135677	day1/pre-b	0.455	0.0019	44
IFN-γ/IL-5 SFC pre-b	SLAMF8_ENSG00000158714	day1/pre-b	0.438	0.0021	47
IFN-γ/IL-5 SFC day 28	PRCP_ENSG00000137509	day1/pre-b	0.45	0.0022	44
IFN-γ/IL-5 SFC day 28	HLA.DMB_ENSG00000242574	day1/pre-b	0.45	0.0022	44
IFN-γ/IL-5 SFC day 28	MVP_ENSG00000013364	day1/pre-b	0.449	0.0022	44

IFN-γ/IL-5 SFC day 28	LIMK2_ENSG00000182541	day1/pre-b	0.449	0.0022	44
IFN-γ/IL-5 SFC pre-b	GBP1_ENSG00000117228	day1/pre-b	0.433	0.0023	47
IFN-γ/IL-5 SFC day 28	X_ENSG00000258581	day1/pre-b	0.446	0.0024	44
IFN-γ/IL-5 SFC day 28	RPN1_ENSG00000163902	day1/pre-b	0.444	0.0025	44
IFN-γ/IL-5 SFC pre-b	CYLD.AS1_ENSG00000261644	day1/pre-b	0.43	0.0025	47
IFN-γ/IL-5 SFC day 28	AKR1A1_ENSG00000117448	day1/pre-b	0.443	0.0026	44
IFN-γ/IL-5 SFC day 28	SLC35A4_ENSG00000176087	day1/pre-b	0.441	0.0027	44
IFN-γ/IL-5 SFC day 28	TCN2_ENSG00000185339	day1/pre-b	0.441	0.0027	44
IFN-γ/IL-5 SFC day 28	STAT2_ENSG00000170581	day1/pre-b	0.44	0.0028	44
IFN-γ/IL-5 SFC day 28	CASP9_ENSG00000132906	day1/pre-b	0.437	0.003	44
IFN-γ/IL-5 SFC pre-b	PSMB9_ENSG00000240065	day1/pre-b	0.423	0.003	47
IFN-γ/IL-5 SFC day 28	TPI1_ENSG00000111669	day1/pre-b	0.436	0.0031	44
IFN-γ/IL-5 SFC day 28	NUCB1_ENSG00000104805	day1/pre-b	0.434	0.0032	44
IFN-γ/IL-5 SFC day 28	PSAP_ENSG00000197746	day1/pre-b	0.434	0.0032	44
IFN-γ/IL-5 SFC day 28	IRF8_ENSG00000140968	day1/pre-b	0.434	0.0033	44
IFN-γ/IL-5 SFC day 28	APOL6_ENSG00000221963	day1/pre-b	0.432	0.0034	44
IFN-γ/IL-5 SFC day 28	HLA.DQB1_ENSG00000179344	day1/pre-b	0.432	0.0034	44
IFN-γ/IL-5 SFC day 28	LSP1_ENSG00000130592	day1/pre-b	0.43	0.0036	44
IFN-γ/IL-5 SFC pre-b	TRIM22_ENSG00000132274	day1/pre-b	0.417	0.0036	47
IFN-γ/IL-5 SFC day 28	ENO1_ENSG00000074800	day1/pre-b	0.429	0.0037	44
IFN-γ/IL-5 SFC day 28	X_ENSG00000271737	day1/pre-b	-0.428	0.0037	44
IFN-γ/IL-5 SFC day 28	CFL1_ENSG00000172757	day1/pre-b	0.427	0.0038	44
IFN-γ/IL-5 SFC day 28	RAB20_ENSG00000139832	day1/pre-b	0.426	0.0039	44
IFN-γ/IL-5 SFC pre-b	FAS_ENSG00000026103	day1/pre-b	0.411	0.0041	47
IFN-γ/IL-5 SFC day 28	CNDP2_ENSG00000133313	day1/pre-b	0.423	0.0043	44
IFN-γ/IL-5 SFC pre-b	APOL1_ENSG00000100342	day1/pre-b	0.408	0.0044	47
IFN-γ/IL-5 SFC day 28	ADA2_ENSG00000093072	day1/pre-b	0.42	0.0045	44
IFN-γ/IL-5 SFC day 28	ZBP1_ENSG00000124256	day1/pre-b	0.42	0.0045	44
IFN-γ/IL-5 SFC day 28	CLK2_ENSG00000176444	day7/pre-b	-0.419	0.0046	44
IFN-γ/IL-5 SFC day 28	TOM1_ENSG00000100284	day1/pre-b	0.418	0.0047	44
IFN-γ/IL-5 SFC day 28	HDGFL2_ENSG00000167674	day7/pre-b	-0.418	0.0047	44
IFN-γ/IL-5 SFC day 28	C1QA_ENSG00000173372	day1/pre-b	0.417	0.0049	44
IFN-γ/IL-5 SFC day 28	ARF3_ENSG00000134287	day1/pre-b	0.416	0.0049	44
IFN-γ/IL-5 SFC day 28	BSG_ENSG00000172270	day1/pre-b	0.416	0.005	44
IFN-γ/IL-5 SFC day 28	PARVG_ENSG00000138964	day1/pre-b	0.413	0.0053	44
IFN-γ/IL-5 SFC day 28	DESI1_ENSG00000100418	day1/pre-b	0.413	0.0053	44
IFN-γ/IL-5 SFC day 28	CD74_ENSG00000019582	day1/pre-b	0.413	0.0054	44
IFN-γ/IL-5 SFC day 28	IDO1_ENSG00000131203	day1/pre-b	0.412	0.0055	44
IFN-γ/IL-5 SFC pre-b	MYBL1_ENSG00000185697	day14/pre-b	0.398	0.0056	47
IFN-γ/IL-5 SFC day 28	LILRB1_ENSG00000104972	day1/pre-b	0.41	0.0057	44
IFN-γ/IL-5 SFC pre-b	GBP4_ENSG00000162654	day1/pre-b	0.397	0.0058	47

IFN-γ/IL-5 SFC day 28	POLR2E_ENSG00000099817	day1/pre-b	0.408	0.0059	44
IFN-γ/IL-5 SFC day 28	UBE2L6_ENSG00000156587	day1/pre-b	0.407	0.0061	44
IFN-γ/IL-5 SFC day 28	PLD3_ENSG00000105223	day1/pre-b	0.407	0.0061	44
IFN-γ/IL-5 SFC day 28	NAPA_ENSG00000105402	day1/pre-b	0.406	0.0063	44
IFN-γ/IL-5 SFC day 28	TNFSF13_ENSG00000161955	day1/pre-b	0.405	0.0063	44
IFN-γ/IL-5 SFC day 28	IFI35_ENSG00000068079	day1/pre-b	0.405	0.0064	44
IFN-γ/IL-5 SFC pre-b	IGHE_ENSG00000211891	day7/pre-b	-0.391	0.0066	47
IFN-γ/IL-5 SFC pre-b	FAM30A_ENSG00000226777	day7/pre-b	-0.391	0.0066	47
IFN-γ/IL-5 SFC day 28	HLA.DRB1_ENSG00000196126	day1/pre-b	0.402	0.0068	44
IFN-γ/IL-5 SFC day 28	PLEK_ENSG00000115956	day1/pre-b	0.402	0.0068	44
IFN-γ/IL-5 SFC day 28	LRG1_ENSG00000171236	day1/pre-b	0.402	0.0069	44
IFN-γ/IL-5 SFC day 28	IRF9_ENSG00000213928	day1/pre-b	0.401	0.007	44
IFN-γ/IL-5 SFC day 28	EIF4E2_ENSG00000135930	day1/pre-b	0.4	0.0071	44
IFN-γ/IL-5 SFC day 28	SEMA4A_ENSG00000196189	day1/pre-b	0.4	0.0071	44
IFN-γ/IL-5 SFC day 28	EAF1_ENSG00000144597	day1/pre-b	0.4	0.0072	44
IFN-γ/IL-5 SFC day 28	FBXO6_ENSG00000116663	day1/pre-b	0.4	0.0072	44
IFN-γ/IL-5 SFC pre-b	IRF9_ENSG00000213928	day1/pre-b	0.387	0.0073	47
IFN-γ/IL-5 SFC day 28	CD63_ENSG00000135404	day1/pre-b	0.397	0.0076	44
IFN-γ/IL-5 SFC day 28	DAZAP2_ENSG00000183283	day1/pre-b	0.397	0.0076	44
IFN-γ/IL-5 SFC day 28	PSMB2_ENSG00000126067	day1/pre-b	0.396	0.0078	44
IFN-γ/IL-5 SFC day 28	LAP3_ENSG00000002549	day1/pre-b	0.395	0.0079	44
IFN-γ/IL-5 SFC day 28	SQOR_ENSG00000137767	day1/pre-b	0.394	0.0081	44
IFN-γ/IL-5 SFC day 28	PTPA_ENSG00000119383	day1/pre-b	0.394	0.0082	44
IFN-γ/IL-5 SFC day 28	TAPBPL_ENSG00000139192	day1/pre-b	0.393	0.0083	44
IFN-γ/IL-5 SFC day 28	FANCE_ENSG00000112039	day14/pre-b	-0.393	0.0084	44
IFN-γ/IL-5 SFC day 28	SZRD1_ENSG00000055070	day1/pre-b	0.391	0.0086	44
IFN-γ/IL-5 SFC day 28	SHKBP1_ENSG00000160410	day1/pre-b	0.391	0.0087	44
IFN-γ/IL-5 SFC day 28	APOL1_ENSG00000100342	day1/pre-b	0.389	0.009	44
IFN-γ/IL-5 SFC day 28	GSN_ENSG00000148180	day1/pre-b	0.389	0.009	44
IFN-γ/IL-5 SFC day 28	TGOLN2_ENSG00000152291	day1/pre-b	0.389	0.0091	44
IFN-γ/IL-5 SFC day 28	NUP93_ENSG00000102900	day1/pre-b	0.389	0.0091	44
IFN-γ/IL-5 SFC day 28	SHTN1_ENSG00000187164	day1/pre-b	0.388	0.0092	44
IFN-γ/IL-5 SFC day 28	GRN_ENSG00000030582	day1/pre-b	0.388	0.0093	44
IFN-γ/IL-5 SFC day 28	CTSH_ENSG00000103811	day1/pre-b	0.388	0.0093	44
IFN-γ/IL-5 SFC pre-b	SERPING1_ENSG00000149131	day1/pre-b	0.375	0.0093	47
IFN-γ/IL-5 SFC day 28	ACLY_ENSG00000131473	day1/pre-b	0.388	0.0093	44
IFN-γ/IL-5 SFC day 28	LGALS9_ENSG00000168961	day1/pre-b	0.388	0.0093	44
IFN-γ/IL-5 SFC day 28	GABARAP_ENSG00000170296	day1/pre-b	0.387	0.0094	44
IFN-γ/IL-5 SFC day 28	PSMD2_ENSG00000175166	day1/pre-b	0.387	0.0094	44
IFN-γ/IL-5 SFC day 28	UBE2D3_ENSG00000109332	day1/pre-b	0.386	0.0096	44
IFN-γ/IL-5 SFC day 28	RAB7A_ENSG00000075785	day1/pre-b	0.386	0.0097	44

IFN-γ/IL-5 SFC day 28	NAGA_ENSG00000198951	day1/pre-b	0.385	0.0099	44
IFN-γ/IL-5 SFC day 28	DBNL_ENSG00000136279	day1/pre-b	0.384	0.01	44
IFN-γ/IL-5 SFC day 28	LILRB4_ENSG00000186818	day1/pre-b	0.384	0.0101	44
IFN-γ/IL-5 SFC pre-b	IGKV2OR22.4_ENSG00000253691	day7/pre-b	-0.371	0.0103	47
IFN-γ/IL-5 SFC day 28	JARID2_ENSG00000008083	day1/pre-b	0.382	0.0105	44
IFN-γ/IL-5 SFC day 28	RNF26_ENSG00000173456	day1/pre-b	0.381	0.0106	44
IFN-γ/IL-5 SFC day 28	DTX3L_ENSG00000163840	day1/pre-b	0.381	0.0108	44
IFN-γ/IL-5 SFC day 28	FXR2_ENSG00000129245	day7/pre-b	-0.381	0.0108	44
IFN-γ/IL-5 SFC day 28	MELK_ENSG00000165304	day7/pre-b	0.381	0.0108	44
IFN-γ/IL-5 SFC pre-b	HLA.DRB5_ENSG00000198502	day1/pre-b	0.368	0.0108	47
IFN-γ/IL-5 SFC day 28	FAM53C_ENSG00000120709	day1/pre-b	0.38	0.0109	44
IFN-γ/IL-5 SFC day 28	EIF4H_ENSG00000106682	day1/pre-b	0.38	0.011	44
IFN-γ/IL-5 SFC day 28	PEPD_ENSG00000124299	day1/pre-b	0.38	0.011	44
IFN-γ/IL-5 SFC day 28	MYOF_ENSG00000138119	day1/pre-b	0.379	0.0111	44
IFN-γ/IL-5 SFC day 28	CUL1_ENSG00000055130	day1/pre-b	0.379	0.0113	44
IFN-γ/IL-5 SFC day 28	PTPN6_ENSG00000111679	day1/pre-b	0.378	0.0115	44
IFN-γ/IL-5 SFC day 28	IGHV2.5_ENSG00000211937	day7/pre-b	0.377	0.0115	44
IFN-γ/IL-5 SFC day 28	BISPR_ENSG00000282851	day1/pre-b	0.377	0.0116	44
IFN-γ/IL-5 SFC pre-b	PSME2_ENSG00000100911	day1/pre-b	0.364	0.0118	47
IFN-γ/IL-5 SFC day 28	TFE3_ENSG00000068323	day1/pre-b	0.376	0.0118	44
IFN-γ/IL-5 SFC day 28	SLC31A1_ENSG00000136868	day1/pre-b	0.376	0.0118	44
IFN-γ/IL-5 SFC day 28	IGHE_ENSG00000211891	day7/pre-b	-0.376	0.0119	44
IFN-γ/IL-5 SFC day 28	WARS1_ENSG00000140105	day1/pre-b	0.376	0.012	44
IFN-γ/IL-5 SFC day 28	MPEG1_ENSG00000197629	day1/pre-b	0.376	0.012	44
IFN-γ/IL-5 SFC day 28	PGAM1_ENSG00000171314	day1/pre-b	0.376	0.012	44
IFN-γ/IL-5 SFC pre-b	UBE2L6_ENSG00000156587	day1/pre-b	0.363	0.012	47
IFN-γ/IL-5 SFC day 28	MAPK1IP1L_ENSG00000168175	day1/pre-b	0.375	0.0121	44
IFN-γ/IL-5 SFC day 28	MYD88_ENSG00000172936	day1/pre-b	0.375	0.0122	44
IFN-γ/IL-5 SFC day 28	AAR2_ENSG00000131043	day1/pre-b	0.375	0.0122	44
IFN-γ/IL-5 SFC day 28	UPP1_ENSG00000183696	day1/pre-b	0.375	0.0122	44
IFN-γ/IL-5 SFC day 28	SERPING1_ENSG00000149131	day1/pre-b	0.374	0.0123	44
IFN-γ/IL-5 SFC day 28	NECAP1_ENSG00000089818	day1/pre-b	0.374	0.0124	44
IFN-γ/IL-5 SFC pre-b	FBXO6_ENSG00000116663	day1/pre-b	0.361	0.0126	47
IFN-γ/IL-5 SFC pre-b	IGLV2.8_ENSG00000278196	day7/pre-b	-0.361	0.0127	47
IFN-γ/IL-5 SFC day 28	ANKRD22_ENSG00000152766	day1/pre-b	0.372	0.013	44
IFN-γ/IL-5 SFC day 28	NLRC5_ENSG00000140853	day1/pre-b	0.371	0.0132	44
IFN-γ/IL-5 SFC day 28	SLC6A12_ENSG00000111181	day1/pre-b	0.371	0.0133	44
IFN-γ/IL-5 SFC pre-b	IRF1_ENSG00000125347	day1/pre-b	0.358	0.0134	47
IFN-γ/IL-5 SFC day 28	TRIM26_ENSG00000234127	day1/pre-b	0.37	0.0135	44
IFN-γ/IL-5 SFC day 28	TNFRSF10B_ENSG00000120889	day1/pre-b	0.37	0.0135	44
IFN-γ/IL-5 SFC day 28	AOAH_ENSG00000136250	day1/pre-b	0.369	0.0137	44

IFN-γ/IL-5 SFC day 28	X_ENSG00000261025	day1/pre-b	-0.367	0.0143	44
IFN-γ/IL-5 SFC day 28	WBP11_ENSG00000084463	day1/pre-b	0.367	0.0143	44
IFN-γ/IL-5 SFC day 28	RNH1_ENSG00000023191	day1/pre-b	0.367	0.0144	44
IFN-γ/IL-5 SFC pre-b	HLA.DPA1_ENSG00000231389	day1/pre-b	0.354	0.0146	47
IFN-γ/IL-5 SFC day 28	NELFB_ENSG00000188986	day7/pre-b	-0.366	0.0146	44
IFN-γ/IL-5 SFC day 28	KXD1_ENSG00000105700	day1/pre-b	0.365	0.0147	44
IFN-γ/IL-5 SFC day 28	LCP1_ENSG00000136167	day1/pre-b	0.365	0.0148	44
IFN-γ/IL-5 SFC day 28	KLF10_ENSG00000155090	day1/pre-b	0.365	0.0148	44
IFN-γ/IL-5 SFC pre-b	GSDMD_ENSG00000104518	day1/pre-b	0.353	0.0148	47
IFN-γ/IL-5 SFC pre-b	HLA.DRA_ENSG00000204287	day1/pre-b	0.353	0.0151	47
IFN-γ/IL-5 SFC day 28	GYG1_ENSG00000163754	day1/pre-b	0.364	0.0152	44
IFN-γ/IL-5 SFC pre-b	GAB1_ENSG00000109458	day7/pre-b	0.351	0.0154	47
IFN-γ/IL-5 SFC pre-b	ANKRD22_ENSG00000152766	day1/pre-b	0.351	0.0155	47
IFN-γ/IL-5 SFC day 28	FCGRT_ENSG00000104870	day1/pre-b	0.363	0.0156	44
IFN-γ/IL-5 SFC day 28	LINC00570_ENSG00000224177	day7/pre-b	-0.361	0.0159	44
IFN-γ/IL-5 SFC pre-b	RIOK3_ENSG00000101782	day7/pre-b	0.35	0.016	47
IFN-γ/IL-5 SFC day 28	RAB5C_ENSG00000108774	day1/pre-b	0.361	0.0161	44
IFN-γ/IL-5 SFC day 28	PPCDC_ENSG00000138621	day1/pre-b	0.361	0.0162	44
IFN-γ/IL-5 SFC pre-b	ETV7_ENSG00000010030	day1/pre-b	0.349	0.0162	47
IFN-γ/IL-5 SFC day 28	IL1RN_ENSG00000136689	day1/pre-b	0.36	0.0163	44
IFN-γ/IL-5 SFC day 28	SLC3A2_ENSG00000168003	day1/pre-b	0.36	0.0164	44
IFN-γ/IL-5 SFC day 28	STXBP2_ENSG00000076944	day1/pre-b	0.36	0.0164	44
IFN-γ/IL-5 SFC day 28	PSMB6_ENSG00000142507	day1/pre-b	0.36	0.0165	44
IFN-γ/IL-5 SFC day 28	VPS18_ENSG00000104142	day1/pre-b	0.359	0.0166	44
IFN-γ/IL-5 SFC day 28	SHISA5_ENSG00000164054	day1/pre-b	0.359	0.0167	44
IFN-γ/IL-5 SFC day 28	JMJD6_ENSG00000070495	day1/pre-b	0.359	0.0167	44
IFN-γ/IL-5 SFC day 28	NA_ENSG00000112096	day1/pre-b	0.359	0.0167	44
IFN-γ/IL-5 SFC day 28	SDHA_ENSG00000073578	day1/pre-b	0.358	0.0169	44
IFN-γ/IL-5 SFC pre-b	GLS_ENSG00000115419	day14/pre-b	0.347	0.017	47
IFN-γ/IL-5 SFC day 28	FCN1_ENSG00000085265	day1/pre-b	0.358	0.017	44
IFN-γ/IL-5 SFC day 28	PGK1_ENSG00000102144	day1/pre-b	0.358	0.017	44
IFN-γ/IL-5 SFC pre-b	BDNF_ENSG00000176697	day7/pre-b	0.346	0.0172	47
IFN-γ/IL-5 SFC day 28	CTSB_ENSG00000164733	day1/pre-b	0.357	0.0173	44
IFN-γ/IL-5 SFC day 28	CAP1_ENSG00000131236	day1/pre-b	0.357	0.0173	44
IFN-γ/IL-5 SFC pre-b	PSMB10_ENSG00000205220	day1/pre-b	0.346	0.0174	47
IFN-γ/IL-5 SFC day 28	RPL32P11_ENSG00000213872	day1/pre-b	-0.356	0.0177	44
IFN-γ/IL-5 SFC day 28	DRAP1_ENSG00000175550	day1/pre-b	0.356	0.0177	44
IFN-γ/IL-5 SFC day 28	HSD3B7_ENSG00000099377	day1/pre-b	0.356	0.0178	44
IFN-γ/IL-5 SFC day 28	JDP2_ENSG00000140044	day1/pre-b	0.355	0.0179	44
IFN-γ/IL-5 SFC pre-b	LAP3_ENSG00000002549	day1/pre-b	0.344	0.0179	47
IFN-γ/IL-5 SFC pre-b	REC8_ENSG00000100918	day1/pre-b	0.344	0.018	47

IFN-γ/IL-5 SFC day 28	ZNF181_ENSG00000197841	day1/pre-b	-0.355	0.018	44
IFN-γ/IL-5 SFC pre-b	EPSTI1_ENSG00000133106	day1/pre-b	0.343	0.0184	47
IFN-γ/IL-5 SFC pre-b	HLA.DRB1_ENSG00000196126	day1/pre-b	0.343	0.0184	47
IFN-γ/IL-5 SFC day 28	TRAFD1_ENSG00000135148	day1/pre-b	0.354	0.0184	44
IFN-γ/IL-5 SFC day 28	BAK1_ENSG00000030110	day1/pre-b	0.354	0.0184	44
IFN-γ/IL-5 SFC pre-b	BATF2_ENSG00000168062	day1/pre-b	0.342	0.0185	47
IFN-γ/IL-5 SFC pre-b	PLK1_ENSG00000166851	day7/pre-b	-0.342	0.0186	47
IFN-γ/IL-5 SFC day 28	ITGB2_ENSG00000160255	day1/pre-b	0.353	0.0186	44
IFN-γ/IL-5 SFC day 28	LCP2_ENSG00000043462	day1/pre-b	0.353	0.0186	44
IFN-γ/IL-5 SFC day 28	DCP1A_ENSG00000272886	day1/pre-b	0.353	0.0186	44
IFN-γ/IL-5 SFC day 28	SEC14L1_ENSG00000129657	day1/pre-b	0.352	0.019	44
IFN-γ/IL-5 SFC day 28	OGDH_ENSG00000105953	day1/pre-b	0.352	0.0191	44
IFN-γ/IL-5 SFC day 28	X_ENSG00000249138	day1/pre-b	0.352	0.0191	44
IFN-γ/IL-5 SFC day 28	NQO2_ENSG00000124588	day1/pre-b	0.352	0.0192	44
IFN-γ/IL-5 SFC pre-b	PSMB3_ENSG00000277791	day1/pre-b	0.34	0.0193	47
IFN-γ/IL-5 SFC day 28	SECTM1_ENSG00000141574	day1/pre-b	0.352	0.0193	44
IFN-γ/IL-5 SFC day 28	FGR_ENSG00000000938	day1/pre-b	0.352	0.0193	44
IFN-γ/IL-5 SFC day 28	APOBEC3A_ENSG00000128383	day1/pre-b	0.351	0.0194	44
IFN-γ/IL-5 SFC pre-b	DNAI4_ENSG00000152763	day1/pre-b	0.339	0.0199	47
IFN-γ/IL-5 SFC day 28	LDHA_ENSG00000134333	day1/pre-b	0.35	0.02	44
IFN-γ/IL-5 SFC day 28	FARP2_ENSG00000006607	day1/pre-b	0.35	0.02	44
IFN-γ/IL-5 SFC pre-b	ORAI3_ENSG00000175938	day7/pre-b	-0.338	0.02	47
IFN-γ/IL-5 SFC pre-b	ZNF567.DT_ENSG00000225975	day1/pre-b	0.338	0.0201	47
IFN-γ/IL-5 SFC day 28	LTA4H_ENSG00000111144	day1/pre-b	0.349	0.0201	44
IFN-γ/IL-5 SFC day 28	GK_ENSG00000198814	day1/pre-b	0.349	0.0204	44
IFN-γ/IL-5 SFC day 28	LILRB3_ENSG00000204577	day1/pre-b	0.348	0.0205	44
IFN-γ/IL-5 SFC day 28	FCGR1A_ENSG00000150337	day1/pre-b	0.348	0.0207	44
IFN-γ/IL-5 SFC day 28	GLUL_ENSG00000135821	day1/pre-b	0.347	0.021	44
IFN-γ/IL-5 SFC day 28	DEXI_ENSG00000182108	day7/pre-b	0.347	0.021	44
IFN-γ/IL-5 SFC pre-b	GBP1P1_ENSG00000225492	day1/pre-b	0.336	0.021	47
IFN-γ/IL-5 SFC day 28	PILRA_ENSG00000085514	day1/pre-b	0.346	0.0213	44
IFN-γ/IL-5 SFC day 28	TNFSF13B_ENSG00000102524	day1/pre-b	0.346	0.0214	44
IFN-γ/IL-5 SFC day 28	GBP4_ENSG00000162654	day1/pre-b	0.346	0.0215	44
IFN-γ/IL-5 SFC pre-b	PSME1_ENSG00000092010	day1/pre-b	0.334	0.0217	47
IFN-γ/IL-5 SFC day 28	IGLV3.9_ENSG00000211670	day7/pre-b	0.345	0.0217	44
IFN-γ/IL-5 SFC day 28	HLA.DRA_ENSG00000204287	day1/pre-b	0.345	0.0219	44
IFN-γ/IL-5 SFC day 28	CFP_ENSG00000126759	day1/pre-b	0.345	0.0219	44
IFN-γ/IL-5 SFC day 28	PKM_ENSG00000067225	day1/pre-b	0.345	0.022	44
IFN-γ/IL-5 SFC day 28	PPP4C_ENSG00000149923	day1/pre-b	0.344	0.0222	44
IFN-γ/IL-5 SFC pre-b	CUL1_ENSG00000055130	day1/pre-b	0.333	0.0222	47
IFN-γ/IL-5 SFC day 28	HSPA5_ENSG00000044574	day1/pre-b	0.343	0.0225	44

IFN-γ/IL-5 SFC day 28	TRAPPC14_ENSG00000146826	day7/pre-b	-0.343	0.0226	44
IFN-γ/IL-5 SFC pre-b	PADI6_ENSG00000276747	day1/pre-b	0.332	0.0227	47
IFN-γ/IL-5 SFC day 28	ARF1_ENSG00000143761	day1/pre-b	0.343	0.0227	44
IFN-γ/IL-5 SFC day 28	SIRPG.AS1_ENSG00000237914	day1/pre-b	0.343	0.0227	44
IFN-γ/IL-5 SFC day 28	PSMB3_ENSG00000277791	day1/pre-b	0.343	0.0228	44
IFN-γ/IL-5 SFC pre-b	HLA.DQB1_ENSG00000179344	day1/pre-b	0.331	0.023	47
IFN-γ/IL-5 SFC day 28	MCTP1_ENSG00000175471	day1/pre-b	0.342	0.023	44
IFN-γ/IL-5 SFC day 28	EIF3B_ENSG00000106263	day7/pre-b	-0.342	0.0231	44
IFN-γ/IL-5 SFC day 28	CIBAR1_ENSG00000188343	day1/pre-b	0.342	0.0232	44
IFN-γ/IL-5 SFC pre-b	HBA2_ENSG00000188536	day7/pre-b	0.331	0.0232	47
IFN-γ/IL-5 SFC day 28	AP2M1_ENSG00000161203	day1/pre-b	0.341	0.0233	44
IFN-γ/IL-5 SFC day 28	OTUB1_ENSG00000167770	day1/pre-b	0.341	0.0236	44
IFN-γ/IL-5 SFC pre-b	VAMP5_ENSG00000168899	day1/pre-b	0.329	0.0238	47
IFN-γ/IL-5 SFC pre-b	GK4P_ENSG00000178146	day1/pre-b	0.329	0.0239	47
IFN-γ/IL-5 SFC day 28	PPP1CA_ENSG00000172531	day1/pre-b	0.34	0.024	44
IFN-γ/IL-5 SFC day 28	RBCK1_ENSG00000125826	day1/pre-b	0.34	0.0241	44
IFN-γ/IL-5 SFC day 28	SLC7A7_ENSG00000155465	day1/pre-b	0.338	0.0248	44
IFN-γ/IL-5 SFC day 28	IRF1_ENSG00000125347	day1/pre-b	0.338	0.0248	44
IFN-γ/IL-5 SFC day 28	X_ENSG00000216775	day7/pre-b	0.338	0.025	44
IFN-γ/IL-5 SFC day 28	CAPG_ENSG00000042493	day1/pre-b	0.337	0.0252	44
IFN-γ/IL-5 SFC pre-b	FANCE_ENSG00000112039	day14/pre-b	-0.326	0.0252	47
IFN-γ/IL-5 SFC day 28	CTNNAL1_ENSG00000119326	day7/pre-b	-0.337	0.0254	44
IFN-γ/IL-5 SFC day 28	LAMP2_ENSG00000005893	day1/pre-b	0.337	0.0255	44
IFN-γ/IL-5 SFC day 28	ANXA5_ENSG00000164111	day1/pre-b	0.336	0.0256	44
IFN-γ/IL-5 SFC day 28	CYLD.AS1_ENSG00000261644	day1/pre-b	0.336	0.0256	44
IFN-γ/IL-5 SFC day 28	TMEM179B_ENSG00000185475	day1/pre-b	0.336	0.0257	44
IFN-γ/IL-5 SFC pre-b	MTHFD2_ENSG00000065911	day1/pre-b	0.325	0.0258	47
IFN-γ/IL-5 SFC day 28	SAT2_ENSG00000141504	day1/pre-b	0.336	0.0258	44
IFN-γ/IL-5 SFC day 28	ZNF394_ENSG00000160908	day1/pre-b	0.336	0.0258	44
IFN-γ/IL-5 SFC day 28	CYBB_ENSG00000165168	day1/pre-b	0.336	0.0258	44
IFN-γ/IL-5 SFC day 28	CES1_ENSG00000198848	day1/pre-b	0.336	0.0259	44
IFN-γ/IL-5 SFC pre-b	HCAR2_ENSG00000182782	day1/pre-b	0.325	0.026	47
IFN-γ/IL-5 SFC day 28	ICAM1_ENSG00000090339	day1/pre-b	0.335	0.0262	44
IFN-γ/IL-5 SFC day 28	PSME2_ENSG00000100911	day1/pre-b	0.335	0.0264	44
IFN-γ/IL-5 SFC day 28	LILRA2_ENSG00000239998	day1/pre-b	0.334	0.0266	44
IFN-γ/IL-5 SFC day 28	HCK_ENSG00000101336	day1/pre-b	0.334	0.0269	44
IFN-γ/IL-5 SFC day 28	NMI_ENSG00000123609	day1/pre-b	0.333	0.027	44
IFN-γ/IL-5 SFC day 28	MX2_ENSG00000183486	day1/pre-b	0.333	0.0271	44
IFN-γ/IL-5 SFC day 28	MPZL2_ENSG00000149573	day1/pre-b	0.333	0.0272	44
IFN-γ/IL-5 SFC pre-b	ZNF383_ENSG00000188283	day1/pre-b	0.322	0.0272	47
IFN-γ/IL-5 SFC day 28	CORO1B_ENSG00000172725	day1/pre-b	0.332	0.0275	44

IFN-γ/IL-5 SFC day 28	ADAM17_ENSG00000151694	day1/pre-b	0.332	0.0279	44
IFN-γ/IL-5 SFC day 28	CASP7_ENSG00000165806	day1/pre-b	0.332	0.0279	44
IFN-γ/IL-5 SFC day 28	STAT3_ENSG00000168610	day1/pre-b	0.331	0.028	44
IFN-γ/IL-5 SFC day 28	EIF5A_ENSG00000132507	day1/pre-b	0.331	0.0282	44
IFN-γ/IL-5 SFC day 28	CPPED1_ENSG00000103381	day1/pre-b	0.331	0.0282	44
IFN-γ/IL-5 SFC pre-b	NCBP1_ENSG00000136937	day1/pre-b	0.32	0.0283	47
IFN-γ/IL-5 SFC day 28	DNPEP_ENSG00000123992	day1/pre-b	0.331	0.0284	44
IFN-γ/IL-5 SFC day 28	IFIT3_ENSG00000119917	day1/pre-b	0.33	0.0286	44
IFN-γ/IL-5 SFC pre-b	SEC11C_ENSG00000166562	day7/pre-b	-0.319	0.0287	47
IFN-γ/IL-5 SFC day 28	ALDOA_ENSG00000149925	day1/pre-b	0.33	0.0287	44
IFN-γ/IL-5 SFC day 28	NUB1_ENSG00000013374	day1/pre-b	0.33	0.0287	44
IFN-γ/IL-5 SFC day 28	AGTRAP_ENSG00000177674	day1/pre-b	0.33	0.0287	44
IFN-γ/IL-5 SFC day 28	ADRM1_ENSG00000130706	day1/pre-b	0.33	0.0289	44
IFN-γ/IL-5 SFC pre-b	FAIM_ENSG00000158234	day1/pre-b	-0.319	0.0289	47
IFN-γ/IL-5 SFC day 28	TM9SF4_ENSG00000101337	day1/pre-b	0.33	0.0289	44
IFN-γ/IL-5 SFC day 28	DUSP3_ENSG00000108861	day1/pre-b	0.33	0.0289	44
IFN-γ/IL-5 SFC day 28	DSE_ENSG00000111817	day1/pre-b	0.329	0.0291	44
IFN-γ/IL-5 SFC day 28	PLEKHM2_ENSG00000116786	day1/pre-b	0.329	0.0293	44
IFN-γ/IL-5 SFC day 28	X_ENSG00000276900	day1/pre-b	0.329	0.0295	44
IFN-γ/IL-5 SFC day 28	CTSS_ENSG00000163131	day1/pre-b	0.328	0.0295	44
IFN-γ/IL-5 SFC day 28	CD33_ENSG00000105383	day1/pre-b	0.327	0.03	44
IFN-γ/IL-5 SFC pre-b	MALINC1_ENSG00000245146	day1/pre-b	-0.316	0.0303	47
IFN-γ/IL-5 SFC day 28	BATF3_ENSG00000123685	day1/pre-b	0.327	0.0303	44
IFN-γ/IL-5 SFC day 28	CDKN1A_ENSG00000124762	day1/pre-b	0.326	0.0308	44
IFN-γ/IL-5 SFC day 28	OAS2_ENSG00000111335	day1/pre-b	0.325	0.0313	44
IFN-γ/IL-5 SFC day 28	GAPDH_ENSG00000111640	day1/pre-b	0.325	0.0314	44
IFN-γ/IL-5 SFC pre-b	FXR2_ENSG00000129245	day7/pre-b	-0.314	0.0314	47
IFN-γ/IL-5 SFC day 28	BST2_ENSG00000130303	day1/pre-b	0.324	0.0317	44
IFN-γ/IL-5 SFC day 28	CSF2RB_ENSG00000100368	day1/pre-b	0.324	0.0318	44
IFN-γ/IL-5 SFC day 28	ACP3_ENSG00000014257	day1/pre-b	0.324	0.0319	44
IFN-γ/IL-5 SFC day 28	LILRB2_ENSG00000131042	day1/pre-b	0.323	0.0323	44
IFN-γ/IL-5 SFC day 28	DDO_ENSG00000203797	day1/pre-b	0.323	0.0323	44
IFN-γ/IL-5 SFC day 28	MSR1_ENSG00000038945	day1/pre-b	0.323	0.0324	44
IFN-γ/IL-5 SFC day 28	SAP30L_ENSG00000164576	day7/pre-b	-0.323	0.0324	44
IFN-γ/IL-5 SFC pre-b	TRAV17_ENSG00000211797	day1/pre-b	0.312	0.0325	47
IFN-γ/IL-5 SFC day 28	RNF144B_ENSG00000137393	day1/pre-b	0.323	0.0326	44
IFN-γ/IL-5 SFC day 28	CLIC1_ENSG00000213719	day1/pre-b	0.322	0.0329	44
IFN-γ/IL-5 SFC day 28	ATP6V0D1_ENSG00000159720	day1/pre-b	0.322	0.033	44
IFN-γ/IL-5 SFC pre-b	IFI35_ENSG00000068079	day1/pre-b	0.311	0.0332	47
IFN-γ/IL-5 SFC day 28	RUFY4_ENSG00000188282	day1/pre-b	0.322	0.0333	44
IFN-γ/IL-5 SFC day 28	PLIN3_ENSG00000105355	day1/pre-b	0.321	0.0333	44

IFN-γ/IL-5 SFC pre-b	PSME2P3_ENSG00000248988	day1/pre-b	0.311	0.0335	47
IFN-γ/IL-5 SFC day 28	TMEM150B_ENSG00000180061	day1/pre-b	0.321	0.0335	44
IFN-γ/IL-5 SFC day 28	KIF2C_ENSG00000142945	day7/pre-b	0.321	0.0337	44
IFN-γ/IL-5 SFC pre-b	IGKJ1_ENSG00000211597	day7/pre-b	-0.31	0.0338	47
IFN-γ/IL-5 SFC day 28	LY6E_ENSG00000160932	day1/pre-b	0.321	0.0338	44
IFN-γ/IL-5 SFC day 28	TNFAIP2_ENSG00000185215	day1/pre-b	0.32	0.0343	44
IFN-γ/IL-5 SFC pre-b	HLA.DRB6_ENSG00000229391	day1/pre-b	0.309	0.0347	47
IFN-γ/IL-5 SFC day 28	SRA1_ENSG00000213523	day1/pre-b	0.319	0.0347	44
IFN-γ/IL-5 SFC day 28	NAMPT_ENSG00000105835	day1/pre-b	0.318	0.0353	44
IFN-γ/IL-5 SFC day 28	CALCOCO2_ENSG00000136436	day1/pre-b	0.318	0.0354	44
IFN-γ/IL-5 SFC day 28	SIRPB1_ENSG00000101307	day1/pre-b	0.318	0.0355	44
IFN-γ/IL-5 SFC pre-b	C2_ENSG00000166278	day1/pre-b	0.307	0.0357	47
IFN-γ/IL-5 SFC day 28	HCLS1_ENSG00000180353	day1/pre-b	0.317	0.0358	44
IFN-γ/IL-5 SFC day 28	AHSA2P_ENSG00000173209	day7/pre-b	-0.317	0.0359	44
IFN-γ/IL-5 SFC day 28	X_ENSG00000269981	day1/pre-b	0.317	0.036	44
IFN-γ/IL-5 SFC day 28	G6PD_ENSG00000160211	day1/pre-b	0.317	0.036	44
IFN-γ/IL-5 SFC day 28	GADD45B_ENSG00000099860	day1/pre-b	0.317	0.036	44
IFN-γ/IL-5 SFC day 28	TTC9_ENSG00000133985	day7/pre-b	-0.317	0.036	44
IFN-γ/IL-5 SFC day 28	FERMT3_ENSG00000149781	day1/pre-b	0.317	0.0363	44
IFN-γ/IL-5 SFC day 28	DDX19B_ENSG00000157349	day1/pre-b	0.317	0.0363	44
IFN-γ/IL-5 SFC day 28	PDLIM5_ENSG00000163110	day1/pre-b	0.317	0.0363	44
IFN-γ/IL-5 SFC day 28	PDCD1LG2_ENSG00000197646	day1/pre-b	0.317	0.0363	44
IFN-γ/IL-5 SFC day 28	SLC31A2_ENSG00000136867	day1/pre-b	0.316	0.0364	44
IFN-γ/IL-5 SFC pre-b	HBB_ENSG00000244734	day7/pre-b	0.306	0.0366	47
IFN-γ/IL-5 SFC pre-b	KIF11_ENSG00000138160	day1/pre-b	0.306	0.0366	47
IFN-γ/IL-5 SFC day 28	UCHL1_ENSG00000154277	day7/pre-b	0.316	0.0366	44
IFN-γ/IL-5 SFC day 28	TUBGCP2_ENSG00000130640	day1/pre-b	0.316	0.0369	44
IFN-γ/IL-5 SFC day 28	GNA13_ENSG00000120063	day1/pre-b	0.315	0.0374	44
IFN-γ/IL-5 SFC pre-b	CD40_ENSG00000101017	day1/pre-b	0.305	0.0374	47
IFN-γ/IL-5 SFC day 28	CD38_ENSG00000004468	day7/pre-b	0.315	0.0375	44
IFN-γ/IL-5 SFC day 28	MOV10_ENSG00000155363	day1/pre-b	0.313	0.0383	44
IFN-γ/IL-5 SFC pre-b	X_ENSG00000216775	day7/pre-b	0.303	0.0383	47
IFN-γ/IL-5 SFC day 28	LIPA_ENSG00000107798	day1/pre-b	0.313	0.0385	44
IFN-γ/IL-5 SFC day 28	P2RX1_ENSG00000108405	day1/pre-b	0.313	0.0387	44
IFN-γ/IL-5 SFC pre-b	PPP1R7_ENSG00000115685	day7/pre-b	-0.302	0.0388	47
IFN-γ/IL-5 SFC day 28	NRBP1_ENSG00000115216	day1/pre-b	0.312	0.0389	44
IFN-γ/IL-5 SFC day 28	OAZ2_ENSG00000180304	day1/pre-b	0.312	0.0394	44
IFN-γ/IL-5 SFC day 28	VPS72_ENSG00000163159	day1/pre-b	0.312	0.0395	44
IFN-γ/IL-5 SFC day 28	CSNK1D_ENSG00000141551	day1/pre-b	0.311	0.0396	44
IFN-γ/IL-5 SFC day 28	THEMIS2_ENSG00000130775	day1/pre-b	0.311	0.0397	44
IFN-γ/IL-5 SFC day 28	RAB24_ENSG00000169228	day1/pre-b	0.311	0.0397	44

IFN-γ/IL-5 SFC pre-b	OAS2_ENSG00000111335	day1/pre-b	0.301	0.04	47
IFN-γ/IL-5 SFC day 28	C19orf38_ENSG00000214212	day1/pre-b	0.311	0.04	44
IFN-γ/IL-5 SFC pre-b	PLSCR1_ENSG00000188313	day1/pre-b	0.3	0.0404	47
IFN-γ/IL-5 SFC pre-b	SIMALR_ENSG00000226004	day1/pre-b	0.3	0.0405	47
IFN-γ/IL-5 SFC day 28	P2RX4_ENSG00000135124	day1/pre-b	0.31	0.0409	44
IFN-γ/IL-5 SFC pre-b	ACVR1B_ENSG00000135503	day1/pre-b	-0.299	0.0409	47
IFN-γ/IL-5 SFC day 28	FCAR_ENSG00000186431	day1/pre-b	0.309	0.0414	44
IFN-γ/IL-5 SFC pre-b	MSR1_ENSG00000038945	day1/pre-b	0.299	0.0415	47
IFN-γ/IL-5 SFC day 28	ATP5F1B_ENSG00000110955	day1/pre-b	0.308	0.0417	44
IFN-γ/IL-5 SFC day 28	CHFR_ENSG00000072609	day14/pre-b	0.308	0.0417	44
IFN-γ/IL-5 SFC day 28	LINC02068_ENSG00000223387	day1/pre-b	0.308	0.0417	44
IFN-γ/IL-5 SFC day 28	PSME2P3_ENSG00000248988	day1/pre-b	0.308	0.042	44
IFN-γ/IL-5 SFC pre-b	TIMP2_ENSG00000035862	day1/pre-b	-0.298	0.0421	47
IFN-γ/IL-5 SFC day 28	IGHV1.3_ENSG00000211935	day7/pre-b	0.308	0.0422	44
IFN-γ/IL-5 SFC day 28	STEAP4_ENSG00000127954	day1/pre-b	0.307	0.0425	44
IFN-γ/IL-5 SFC day 28	GLB1_ENSG00000170266	day1/pre-b	0.306	0.0431	44
IFN-γ/IL-5 SFC day 28	HIF1A_ENSG00000100644	day1/pre-b	0.306	0.0431	44
IFN-γ/IL-5 SFC pre-b	SH2D2A_ENSG00000027869	day1/pre-b	0.296	0.0433	47
IFN-γ/IL-5 SFC day 28	GPBAR1_ENSG00000179921	day1/pre-b	0.306	0.0433	44
IFN-γ/IL-5 SFC day 28	PSMB8_ENSG00000204264	day1/pre-b	0.306	0.0433	44
IFN-γ/IL-5 SFC day 28	IL27_ENSG00000197272	day1/pre-b	0.306	0.0435	44
IFN-γ/IL-5 SFC day 28	X_ENSG00000224579	day1/pre-b	0.306	0.0436	44
IFN-γ/IL-5 SFC day 28	DCTN1_ENSG00000204843	day1/pre-b	0.305	0.044	44
IFN-γ/IL-5 SFC day 28	CPQ_ENSG00000104324	day1/pre-b	0.305	0.0442	44
IFN-γ/IL-5 SFC day 28	GAA_ENSG00000171298	day1/pre-b	0.304	0.0448	44
IFN-γ/IL-5 SFC pre-b	TRBV24.1_ENSG00000211750	day1/pre-b	0.294	0.045	47
IFN-γ/IL-5 SFC pre-b	PIM1_ENSG00000137193	day1/pre-b	0.294	0.0451	47
IFN-γ/IL-5 SFC day 28	CAPZB_ENSG00000077549	day1/pre-b	0.304	0.0451	44
IFN-γ/IL-5 SFC day 28	MLF2_ENSG00000089693	day1/pre-b	0.304	0.0451	44
IFN-γ/IL-5 SFC day 28	NLRP3_ENSG00000162711	day1/pre-b	0.303	0.0456	44
IFN-γ/IL-5 SFC day 28	SDE2_ENSG00000143751	day1/pre-b	0.302	0.0463	44
IFN-γ/IL-5 SFC day 28	NANS_ENSG00000095380	day1/pre-b	0.302	0.0465	44
IFN-γ/IL-5 SFC day 28	TTC9_ENSG00000133985	day1/pre-b	-0.302	0.0466	44
IFN-γ/IL-5 SFC pre-b	TAP1_ENSG00000168394	day1/pre-b	0.291	0.0469	47
IFN-γ/IL-5 SFC pre-b	TYMS_ENSG00000176890	day7/pre-b	0.291	0.0472	47
IFN-γ/IL-5 SFC day 28	X_ENSG00000203279	day1/pre-b	-0.301	0.0472	44
IFN-γ/IL-5 SFC day 28	RALB_ENSG00000144118	day1/pre-b	0.3	0.0475	44
IFN-γ/IL-5 SFC pre-b	RHOXF1P1_ENSG00000234493	day7/pre-b	0.29	0.0481	47
IFN-γ/IL-5 SFC pre-b	DEFA3_ENSG00000239839	day7/pre-b	0.29	0.0482	47
IFN-γ/IL-5 SFC day 28	OASL_ENSG00000135114	day1/pre-b	0.299	0.0484	44
IFN-γ/IL-5 SFC day 28	SPIB_ENSG00000269404	day1/pre-b	0.299	0.0485	44

IFN-γ/IL-5 SFC pre-b	X_ENSG00000271737	day1/pre-b	-0.289	0.0486	47
IFN-γ/IL-5 SFC day 28	MGAT1_ENSG00000131446	day1/pre-b	0.299	0.0488	44
IFN-γ/IL-5 SFC day 28	PARP14_ENSG00000173193	day1/pre-b	0.299	0.0488	44
IFN-γ/IL-5 SFC pre-b	MT2A_ENSG00000125148	day1/pre-b	0.289	0.0488	47
IFN-γ/IL-5 SFC pre-b	KARS1_ENSG00000065427	day1/pre-b	0.289	0.0492	47
IFN-γ/IL-5 SFC day 28	REC8_ENSG00000100918	day1/pre-b	0.298	0.0494	44
IFN-γ/IL-5 SFC day 28	SERTAD3_ENSG00000167565	day1/pre-b	0.298	0.0496	44
IFN-γ/IL-5 SFC day 28	APOL3_ENSG00000128284	day1/pre-b	0.298	0.0498	44

Supplementary Table 4. Spearman correlation statistics between plasma cytokine changes (post/pre-b) and T cell polarization (IFN-γ/IL-5 SFC) pre- and 28 days post-booster. Significant correlates are shown (P<0.05).

Parameter 1: Plasma cytokine changes	Parameter 2: Th1 polarization	r	*P*	n
IFNG (d14/pre-b)	IFN-γ/IL-5 SFC day 28	0.453	0.001	47
IL27 (d14/pre-b)	IFN-γ/IL-5 SFC day 28	0.433	0.002	47
CXCL9 (d14/pre-b)	IFN-γ/IL-5 SFC day 28	0.419	0.003	47
CCL4 (d14/pre-b)	IFN-γ/IL-5 SFC day 28	0.388	0.007	47
CXCL11 (d14/pre-b)	IFN-γ/IL-5 SFC day 28	0.350	0.016	47
IFNG (d1/pre-b)	IFN-γ/IL-5 SFC day 28	0.339	0.021	46
HGF (d14/pre-b)	IFN-γ/IL-5 SFC day 28	0.322	0.027	47
FLT3LG (d14/pre-b)	IFN-γ/IL-5 SFC day 28	0.312	0.033	47
IL2 (d3/pre-b)	IFN-γ/IL-5 SFC day 28	0.333	0.034	41
CCL3 (d14/pre-b)	IFN-γ/IL-5 SFC day 28	0.303	0.038	47
OLR1 (d14/pre-b)	IFN-γ/IL-5 SFC day 28	0.299	0.041	47
CXCL11 (d3/pre-b)	IFN-γ/IL-5 SFC day 28	0.300	0.043	46
CXCL9 (d3/pre-b)	IFN-γ/IL-5 SFC day 28	0.294	0.045	47
IL27 (d1/pre-b)	IFN-γ/IL-5 SFC pre-b	0.328	0.023	48
IL27 (d14/pre-b)	IFN-γ/IL-5 SFC pre-b	0.327	0.021	50
CXCL9 (d3/pre-b)	IFN-γ/IL-5 SFC pre-b	0.300	0.034	50
CXCL9 (d14/pre-b)	IFN-γ/IL-5 SFC pre-b	0.331	0.019	50
CCL11 (d14/pre-b)	IFN-γ/IL-5 SFC pre-b	0.380	0.006	50
HGF (d7/pre-b)	IFN-γ/IL-5 SFC pre-b	0.350	0.014	49
CXCL10 (d14/pre-b)	IFN-γ/IL-5 SFC pre-b	0.290	0.041	50
IFNG (d1/pre-b)	IFN-γ/IL-5 SFC pre-b	0.473	0.001	48
IFNG (d3/pre-b)	IFN-γ/IL-5 SFC pre-b	0.329	0.019	50
IFNG (d7/pre-b)	IFN-γ/IL-5 SFC pre-b	0.286	0.046	49
IFNG (d14/pre-b)	IFN-γ/IL-5 SFC pre-b	0.446	0.001	50
TNF (d1/pre-b)	IFN-γ/IL-5 SFC pre-b	0.338	0.019	48
VEGFA (d14/pre-b)	IFN-γ/IL-5 SFC pre-b	0.311	0.028	50
OSM (d7/pre-b)	IFN-γ/IL-5 SFC pre-b	0.299	0.037	49
CCL4 (d7/pre-b)	IFN-γ/IL-5 SFC pre-b	0.331	0.020	49
CCL4 (d14/pre-b)	IFN-γ/IL-5 SFC pre-b	0.320	0.023	50
CXCL11 (d3/pre-b)	IFN-γ/IL-5 SFC pre-b	0.367	0.010	49
CXCL11 (d14/pre-b)	IFN-γ/IL-5 SFC pre-b	0.334	0.018	50

Supplementary Table 5. Antibodies and dye details.

Target	Conjugate	Host	Target	Clone	Catalog	Vendor	Application	Dilution
IgG	PE	Mouse	Human	JDC-10	9040-09	SouthernBiotech	Ab response	1/50
IgG1	PE	Mouse	Human	HP6001	9054-09	SouthernBiotech	Ab response	1/250
IgG2	PE	Mouse	Human	HP6025	9070-09	SouthernBiotech	Ab response	1/50
IgG3	PE	Mouse	Human	HP6050	9210-09	SouthernBiotech	Ab response	1/50
IgG4	PE	Mouse	Human	HP6025	9200-09	SouthernBiotech	Ab response	1/50
CD4	APC-eF780	Mouse	Human	RPA-T4	47-0049-42	LIFE TECH	AIM Assay	1/50
CD3	AF700	Mouse	Human	UCHT1	56-0038-42	eBioscience	AIM Assay	1/50
CD8	V500	Mouse	Human	RPA-T8	560774	BD	AIM Assay	1/100
CD14	V500	Mouse	Human	M5E2	561391	BD	AIM Assay	1/100
CD19	V500	Mouse	Human	HIB19	561121	BD	AIM Assay	1/100
CD45RA	eF450	Mouse	Human	HI100	48-0458-42	Invitrogen	AIM Assay	1/50
CCR7	PerCP-Cy5.5	Mouse	Human	G043H7	353200	Biolegend	AIM Assay	1/25
OX40	PE-Cy7	Mouse	Human	Ber-ACT35	350012	Biolegend	AIM Assay	1/50
CD137	APC	Mouse	Human	4B4-1	309810	Biolegend	AIM Assay	1/50
CD25	FITC	Mouse	Human	M-A251	555431	BD	AIM Assay	1/50
CD69	BV605	Mouse	Human	FN50	562989	BD	AIM Assay	1/50
PDL1	PE	Mouse	Human	29E.2A3	329706	Biolegend	AIM Assay	1/50
Viability Dye	eF506	-	All species	-	65-0866-14	Thermo Fisher	AIM Assay	1/500
