# Supplementary material for: Th1 polarization in Bordetella pertussis vaccine responses is maintained through a positive feedback loop
Source: bioRxiv. 2024 Oct 17:2024.08.05.606623. Originally published 2024 Aug 7. Preprint. [Version 2] doi: 10.1101/2024.08.05.606623 (PMC11326151; doi:10.1101/2024.08.05.606623)
Supplement: Supplement 2 [file NIHPP2024.08.05.606623v2-supplement-2.pdf]

bioRxiv preprint doi: https://doi.org/10.1101/2024.08.05.606623; this version posted October 17, 2024. The copyright holder for this preprint (which was not certified by peer review) is the author/funder, who has granted bioRxiv a license to display the preprint in perpetuity. It is made available under aCC-BY 4.0 International license.

Supplementary Fig. 1

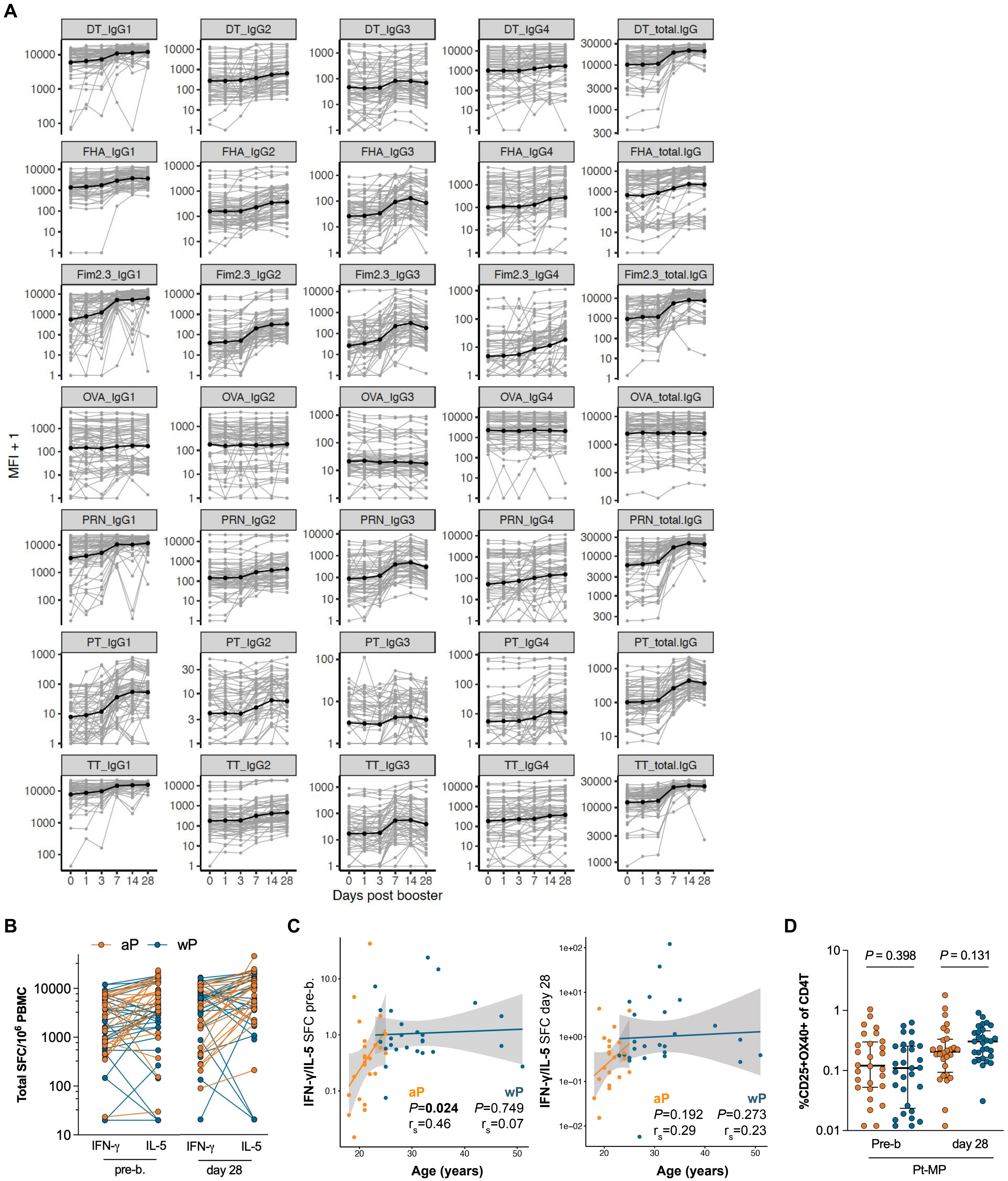

Supplementary Fig. 1. **Whole-cell pertussis vaccine priming increases Th1 polarization despite acellular booster vaccination.** **A)** Plasma IgG measurements before and after Tdap booster vaccination. Shown are $\log_{10}$-scaled median fluorescence intensities (MFI) of IgG1-4 and total IgG against Tdap antigens (PT, PRN, FHA, FIM2/3, TT, and DT) and the non-Tdap antigen ovalbumin (OVA) for each participant (n=57) and time point (grey). The black lines and points indicate the medians. **B**) IFN-γ and IL-5 producing cells (spot-forming cells, SFC) were measured by Fluorospot after cells were stimulated with aP vaccine antigens for 14 days and derived from blood sampled before and 28 days after Tdap booster vaccination. **C)** Spearman sample correlations between age (years) at booster and T cell polarization data before and 28 days post booster (IFN-γ/IL-5 SFC) per vaccination group. **D)** $CD25^{+}$ and $OX40^{+}$ $CD4^{+}$ T cells were measured before and 28 days after Tdap booster vaccination by flow cytometry and shown as percentage of total $CD4^{+}$ T cells where black lines represent the median with interquartile range. n=28 aP, 29 wP, P-values were calculated by multiple two-tailed Mann-Whitney tests.

bioRxiv preprint doi: https://doi.org/10.1101/2024.08.05.606623; this version posted October 17, 2024. The copyright holder for this preprint (which was not certified by peer review) is the author/funder, who has granted bioRxiv a license to display the preprint in perpetuity. It is made available under aCC-BY 4.0 International license.

Supplementary Fig 2

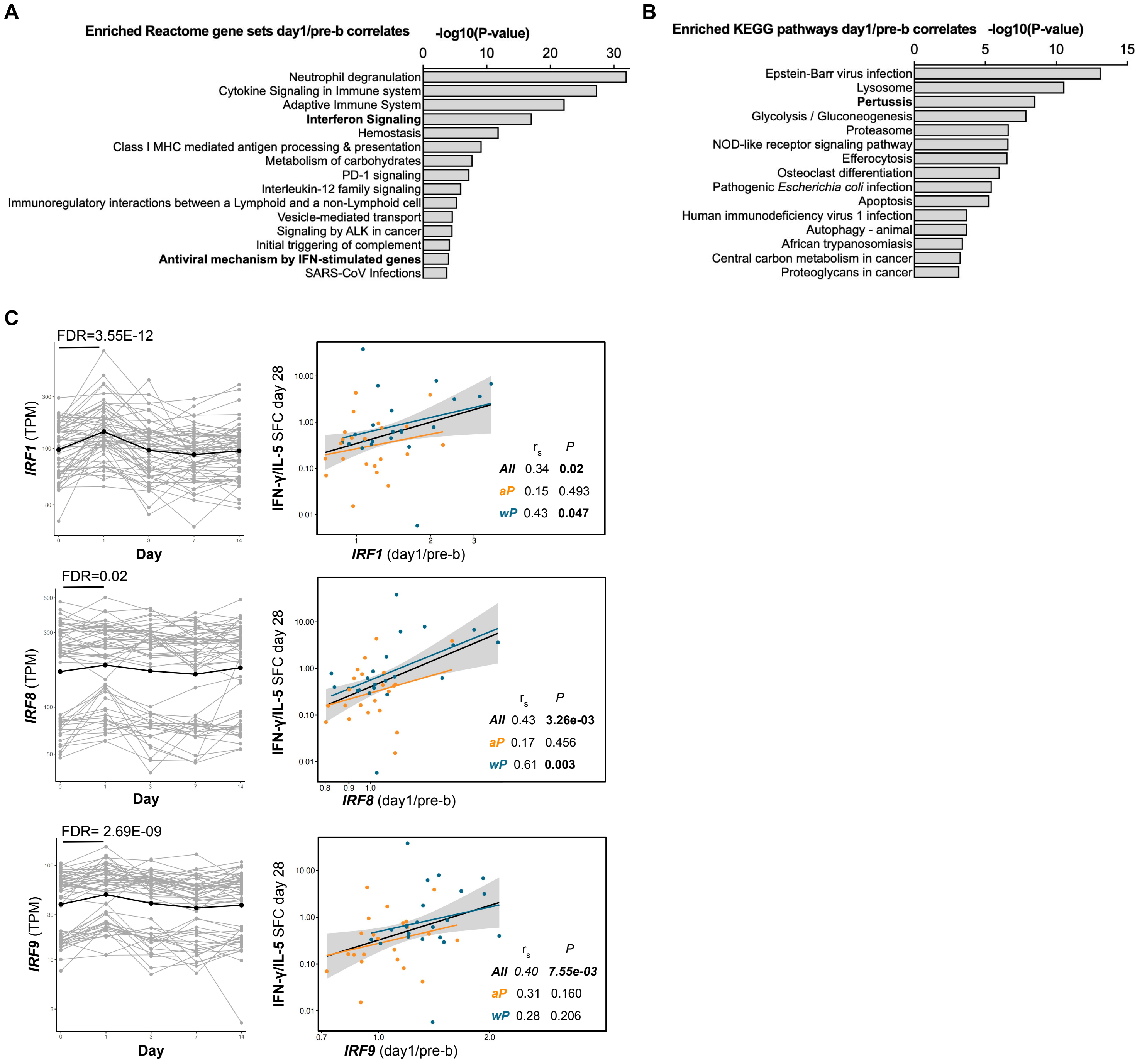

Supplementary Fig. 2. **Booster-induced interferon signaling positively correlates with Th1 polarization.**
A-B) The top 15 enriched gene sets identified by **(A)** Reactome or **(B)** Kyoto Encyclopedia of Genes and Genomes (KEGG) gene set enrichment analysis with the 294 DEG (day 1/pre-booster) that also showed a positive correlation with the T cell polarization on day 28. **C)** *IRF1, IRF8* and *IRF9* gene expression (TPM) over time with the black line indicating mean expression and Spearman sample correlation between *IRF's* (day 1/pre-b) and T cell polarization data 28 days post booster (IFN-γ/IL-5 SFC). Spearman dot plots were created with aP and wP groups combined ("All") and separately. RNA n=56, correlation analysis n=44. **A-C**) aP and wP-primed individuals were pooled for this analysis.

bioRxiv preprint doi: https://doi.org/10.1101/2024.08.05.606623; this version posted October 17, 2024. The copyright holder for this preprint (which was not certified by peer review) is the author/funder, who has granted bioRxiv a license to display the preprint in perpetuity. It is made available under aCC-BY 4.0 International license.

Supplementary Fig. 3

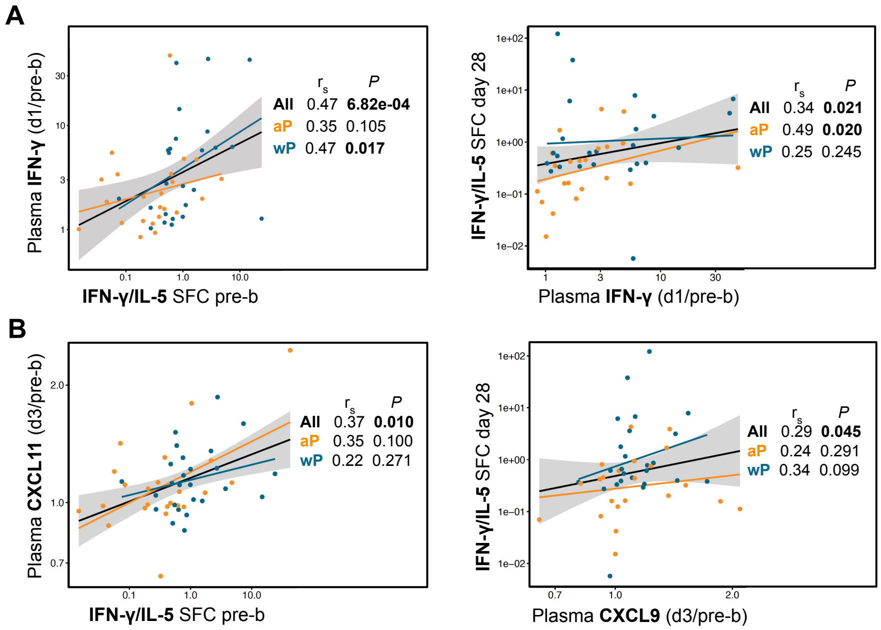

Supplementary Fig. 3. **Plasma IFN-γ and IFN-γ initiated chemokines increase post-booster and its changes positively correlate with Th1 polarization.**

A-B) Spearman sample correlation between plasma cytokine changes and T cell polarization pre- and 28 days post booster (IFN-γ/IL-5 SFC). Spearman dot plots were created with aP and wP groups combined ("All") and separately.

bioRxiv preprint doi: https://doi.org/10.1101/2024.08.05.606623; this version posted October 17, 2024. The copyright holder for this preprint (which was not certified by peer review) is the author/funder, who has granted bioRxiv a license to display the preprint in perpetuity. It is made available under aCC-BY 4.0 International license.

Supplementary Fig. 4

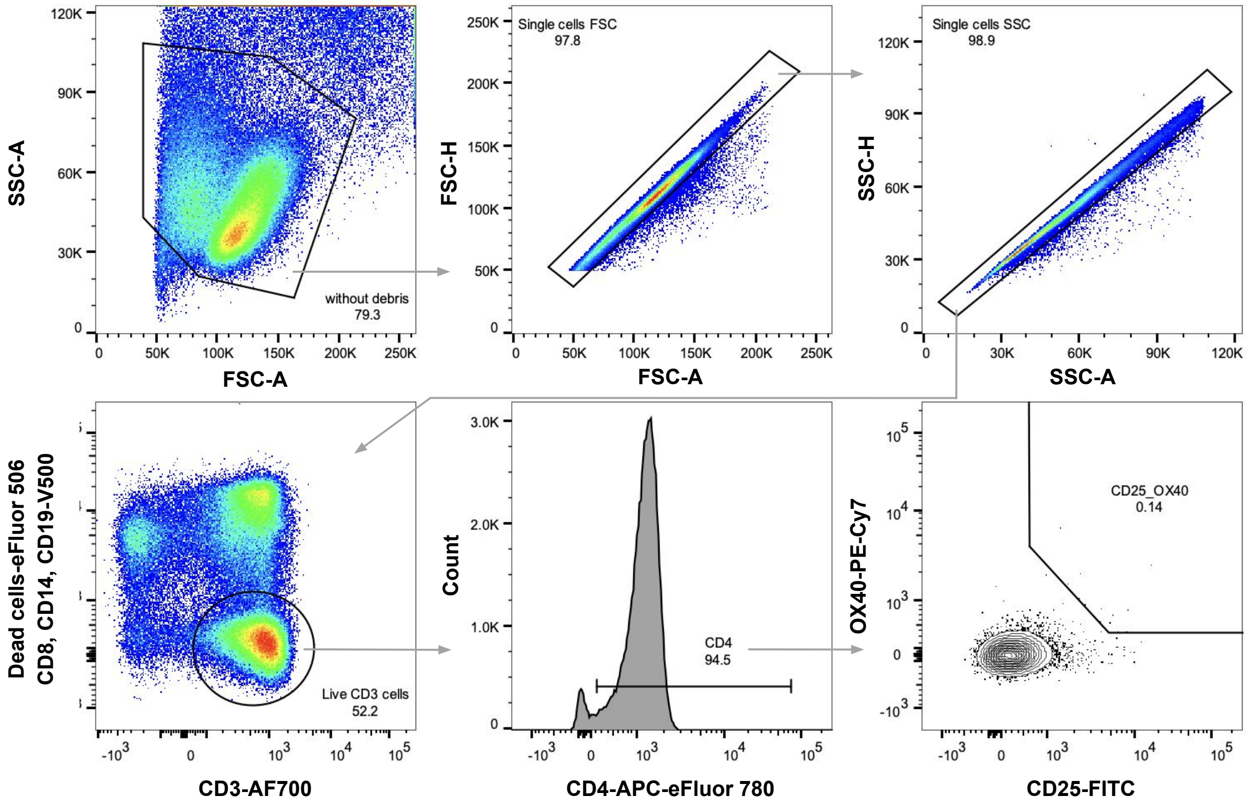

Supplementary Fig. 4. **Strategy for flow cytometry gating AIM CD25$^+$OX40$^+$ CD4$^+$ T cells.**
