# Supplementary material for: Th1 polarization in Bordetella pertussis vaccine responses is maintained through a positive feedback loop
Source: bioRxiv. 2024 Aug 7:2024.08.05.606623. Preprint. [Version 1] doi: 10.1101/2024.08.05.606623 (PMC11326151; doi:10.1101/2024.08.05.606623)
Supplement: Supplementary file 1 [file NIHPP2024.08.05.606623v1-supplement-2.pdf]

bioRxiv preprint doi: https://doi.org/10.1101/2024.08.05.606623; this version posted August 7, 2024. The copyright holder for this preprint (which was not certified by peer review) is the author/funder, who has granted bioRxiv a license to display the preprint in perpetuity. It is made available under aCC-BY 4.0 International license.

Figure S1

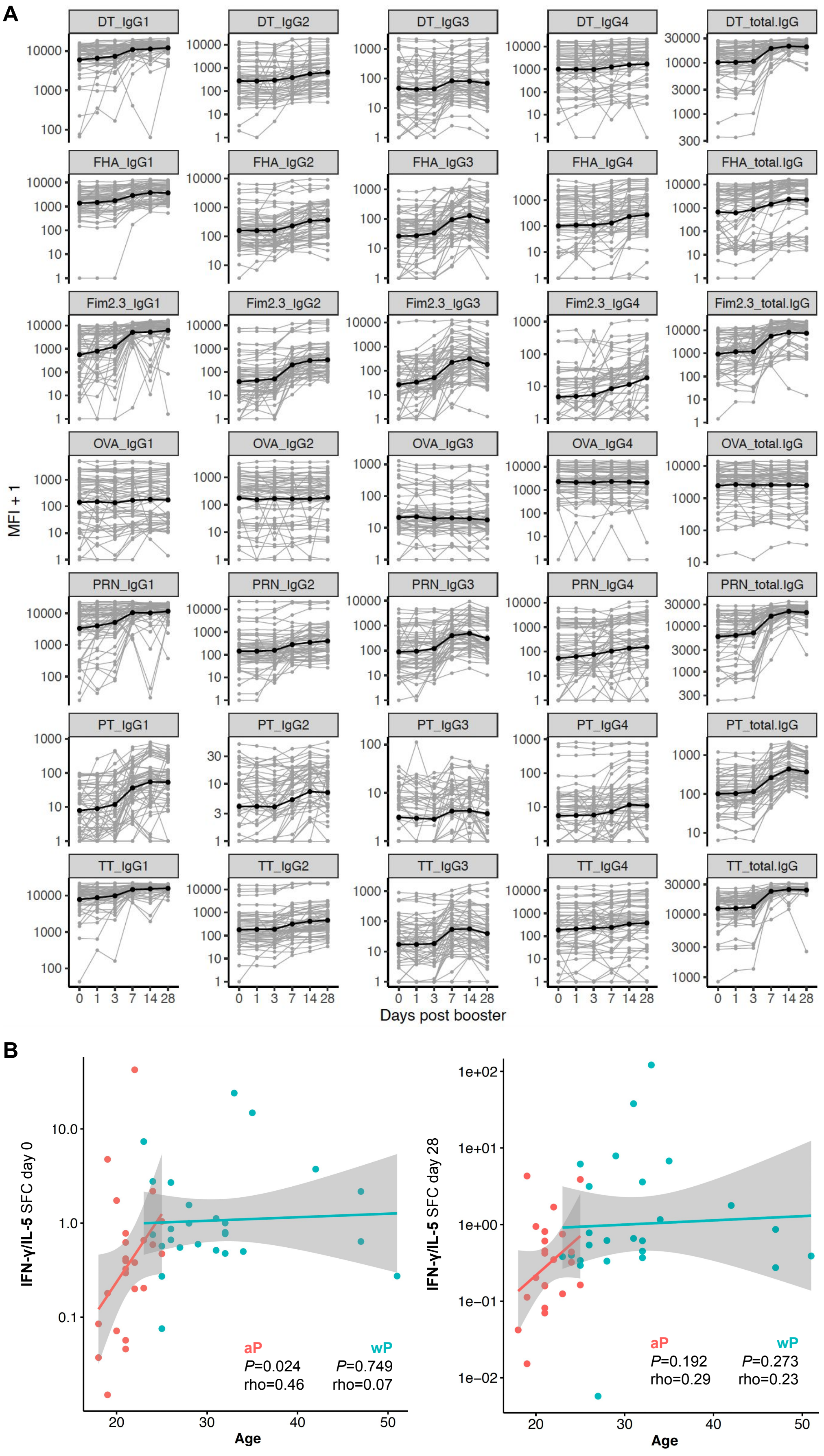

Figure S1. **A)** Plasma IgG measurements before and after Tdap booster vaccination. Shown are $\log_{10}$-scaled median fluorescence intensities (MFI) of IgG1-4 and total IgG against Tdap antigens (PT, PRN, FHA, FIM2/3, TT, and DT) and the non-Tdap antigen ovalbumin (OVA) for each participant (n=57) and time point (grey). The black lines and points indicate the medians. **B)** Spearman correlations between age at booster and T cell polarization data before and 28 days post booster (IFN-γ/IL-5 SFC) per vaccination group.

bioRxiv preprint doi: https://doi.org/10.1101/2024.08.05.606623; this version posted August 7, 2024. The copyright holder for this preprint (which was not certified by peer review) is the author/funder, who has granted bioRxiv a license to display the preprint in perpetuity. It is made available under aCC-BY 4.0 International license.

Figure S2

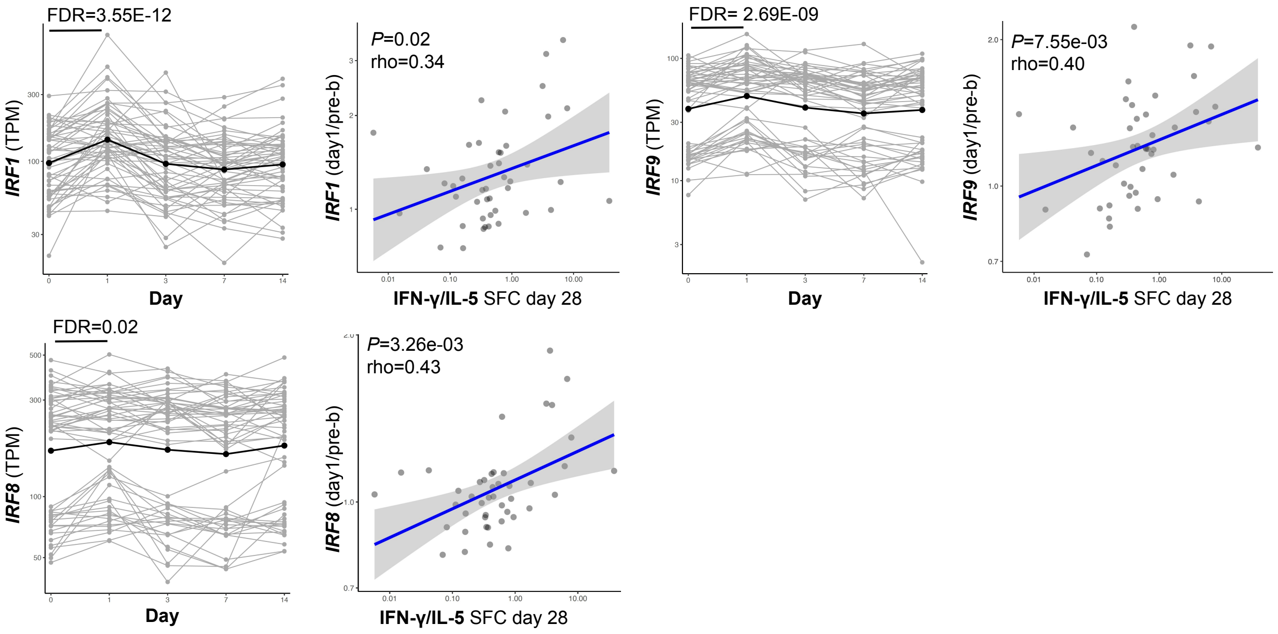

Figure S2. **Interferon signaling positively correlates with Th1 polarization.** *IRF1, IRF8* and *IRF9* gene expression (TPM) over time with the black line indicating mean expression and Spearman correlation between *IRF's* (day 1/pre-b) and T cell polarization data 28 days post booster (IFN-γ/IL-5 SFC). RNA n=56, correlation analysis n=44.

bioRxiv preprint doi: https://doi.org/10.1101/2024.08.05.606623; this version posted August 7, 2024. The copyright holder for this preprint (which was not certified by peer review) is the author/funder, who has granted bioRxiv a license to display the preprint in perpetuity. It is made available under aCC-BY 4.0 International license.

Figure S3

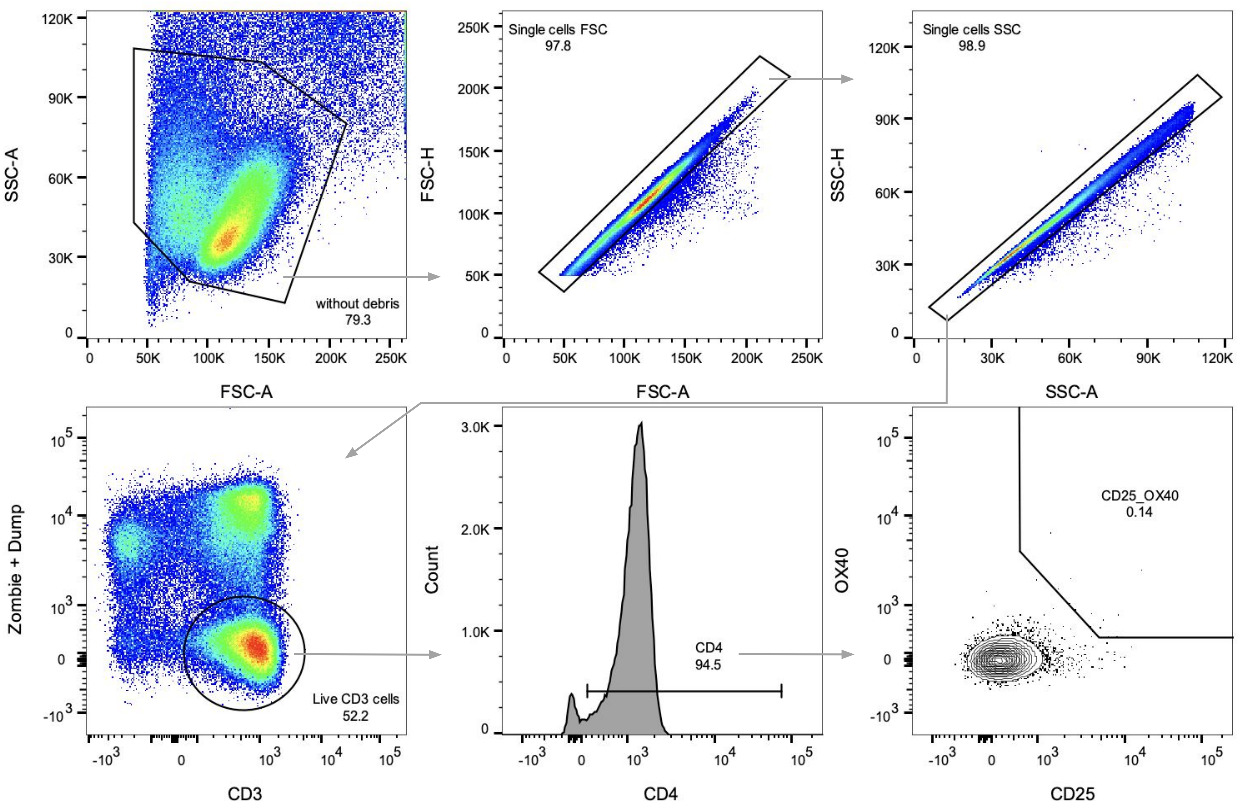

Figure S3. **Strategy for gating AIM CD25$^+$OX40$^+$ CD4$^+$ T cells.** The dump gate consisted of V500-conjugated CD8, CD14, and CD19.
