# Supplementary material for: Th1 polarization in Bordetella pertussis vaccine responses is maintained through a positive feedback loop
Source: bioRxiv. 2024 Aug 7:2024.08.05.606623. Preprint. [Version 1] doi: 10.1101/2024.08.05.606623 (PMC11326151; doi:10.1101/2024.08.05.606623)
Supplement: Supplementary file 2 [file media-1.pdf]

Table S1. Study population characteristics. Shapiro-Wilk and Mann-Whitney tests were performed to test differences in age at booster between the aP and wP groups. A Chi-square test was used to check if biological sex differed per group.

	aP (n=31)	wP (n=30)	*P*-value
**Age at booster, mean years ± SD**	21.1 ± 2.2	31.0 ± 7.2	***P*<0.001**
**Sex, n male (%)**	10 (32.3%)	10 (33.3%)	*P*=0.929

Table S2. Performed assays per donor.

Subject ID	Group	AIM	Fluorospot	Plasma antibodies	Plasma cytokines	RNAseq
2	wP	Yes	Yes	Yes	Yes	NO
97	wP	Yes	Yes	Yes	Yes	Yes
8	wP	Yes	Yes	Yes	Yes	NO
26	wP	NO	NO	NO	Yes	NO
45	aP	NO	NO	NO	Yes	NO
61	wP	Yes	Yes	Yes	Yes	Yes
62	wP	Yes	Yes	Yes	Yes	Yes
63	wP	Yes	Yes	Yes	Yes	Yes
64	wP	Yes	Yes	Yes	Yes	Yes
65	wP	Yes	NO	Yes	Yes	Yes
66	wP	Yes	Yes	Yes	Yes	Yes
67	wP	Yes	Yes	Yes	Yes	Yes
68	wP	Yes	Yes	Yes	Yes	Yes
69	wP	Yes	Yes	Yes	Yes	Yes
70	aP	Yes	Yes	Yes	Yes	Yes
71	aP	Yes	Yes	Yes	Yes	Yes
72	wP	Yes	Yes	Yes	Yes	Yes
73	wP	Yes	Yes	Yes	Yes	Yes
74	wP	Yes	Yes	Yes	Yes	Yes
75	aP	Yes	Yes	Yes	Yes	Yes
76	aP	Yes	Yes	Yes	Yes	Yes
77	wP	Yes	Yes	Yes	Yes	Yes
78	wP	Yes	Yes	Yes	Yes	Yes
79	wP	Yes	Yes	Yes	Yes	Yes
80	wP	Yes	NO	Yes	Yes	Yes
81	wP	Yes	Yes	Yes	Yes	Yes
82	aP	Yes	Yes	NO	Yes	Yes

83	aP	Yes	NO	Yes	Yes	Yes
84	aP	Yes	Yes	Yes	Yes	Yes
85	aP	Yes	NO	Yes	Yes	Yes
86	aP	Yes	Yes	Yes	Yes	Yes
87	aP	NO	NO	Yes	Yes	Yes
88	aP	NO	NO	NO	Yes	Yes
89	aP	Yes	Yes	Yes	Yes	Yes
90	aP	Yes	Yes	Yes	Yes	Yes
91	aP	Yes	Yes	Yes	Yes	Yes
92	aP	Yes	Yes	Yes	Yes	Yes
93	aP	Yes	Yes	Yes	Yes	Yes
94	aP	Yes	Yes	Yes	Yes	Yes
95	aP	Yes	Yes	Yes	Yes	Yes
96	aP	Yes	Yes	Yes	Yes	Yes
98	wP	Yes	Yes	Yes	Yes	Yes
99	aP	Yes	Yes	Yes	Yes	Yes
100	aP	Yes	NO	Yes	Yes	Yes
101	aP	Yes	Yes	Yes	Yes	Yes
102	aP	Yes	Yes	Yes	Yes	Yes
103	wP	Yes	Yes	Yes	Yes	Yes
104	wP	Yes	Yes	Yes	Yes	Yes
105	wP	Yes	NO	Yes	Yes	Yes
106	aP	Yes	Yes	Yes	Yes	Yes
107	aP	Yes	Yes	Yes	Yes	Yes
108	wP	Yes	Yes	Yes	Yes	Yes
109	wP	Yes	Yes	Yes	Yes	Yes
110	aP	Yes	Yes	Yes	Yes	Yes
111	wP	Yes	Yes	Yes	Yes	Yes
112	aP	Yes	Yes	Yes	Yes	Yes

114	wP	Yes	Yes	Yes	Yes	Yes
115	aP	Yes	Yes	Yes	Yes	Yes
117	aP	Yes	NO	Yes	Yes	Yes
118	aP	Yes	Yes	Yes	Yes	Yes
0	wP	Yes	Yes	Yes	Yes	NO

Table S3. Spearman correlation statistics between plasma cytokine changes (post/pre-b) and T cell polarization data 28 days post booster (IFN-γ/IL-5 SFC)

**Parameter2** (Olink)	**Parameter1** (Fluorospot)	**rho**	**P-value**	**n**
**IFNG.d14/pre-b**	**IFNG.IL5.d28**	**0.453**	**0.001**	47
**IL27.d14/pre-b**	**IFNG.IL5.d28**	**0.433**	**0.002**	47
**CXCL9.d14/pre-b**	**IFNG.IL5.d28**	**0.419**	**0.003**	47
**CCL4.d14/pre-b**	**IFNG.IL5.d28**	**0.388**	**0.007**	47
**CXCL11.d14/pre-b**	**IFNG.IL5.d28**	**0.350**	**0.016**	47
**IFNG.d1/pre-b**	**IFNG.IL5.d28**	**0.339**	**0.021**	46
**HGF.d14/pre-b**	**IFNG.IL5.d28**	**0.322**	**0.027**	47
**FLT3LG.d14/pre-b**	**IFNG.IL5.d28**	**0.312**	**0.033**	47
**IL2.d3/pre-b**	**IFNG.IL5.d28**	**0.333**	**0.034**	41
**CCL3.d14/pre-b**	**IFNG.IL5.d28**	**0.303**	**0.038**	47
**OLR1.d14/pre-b**	**IFNG.IL5.d28**	**0.299**	**0.041**	47
**CXCL11.d3/pre-b**	**IFNG.IL5.d28**	**0.300**	**0.043**	46
**CXCL9.d3/pre-b**	**IFNG.IL5.d28**	**0.294**	**0.045**	47
CCL19.d14/pre-b	IFNG.IL5.d28	0.284	0.053	47
MMP12.d14/pre-b	IFNG.IL5.d28	0.283	0.054	47
IL6.d14/pre-b	IFNG.IL5.d28	0.283	0.054	47
CXCL10.d14/pre-b	IFNG.IL5.d28	0.276	0.060	47
TGFA.d14/pre-b	IFNG.IL5.d28	0.276	0.061	47
CXCL8.d14/pre-b	IFNG.IL5.d28	0.270	0.067	47
IL17C.d1/pre-b	IFNG.IL5.d28	-0.270	0.069	46
CCL4.d3/pre-b	IFNG.IL5.d28	0.259	0.079	47
TNFSF12.d14/pre-b	IFNG.IL5.d28	0.256	0.082	47
TNF.d1/pre-b	IFNG.IL5.d28	0.259	0.082	46
IFNG.d3/pre-b	IFNG.IL5.d28	0.253	0.086	47
VEGFA.d14/pre-b	IFNG.IL5.d28	0.253	0.086	47
TNFSF10.d7/pre-b	IFNG.IL5.d28	-0.228	0.127	46
CCL3.d3/pre-b	IFNG.IL5.d28	0.224	0.131	47

CXCL9.d1/pre-b	IFNG.IL5.d28	0.225	0.132	46
TNF.d14/pre-b	IFNG.IL5.d28	0.208	0.161	47
EGF.d14/pre-b	IFNG.IL5.d28	0.205	0.168	47
TGFA.d3/pre-b	IFNG.IL5.d28	0.203	0.170	47
IL15.d14/pre-b	IFNG.IL5.d28	0.203	0.172	47
CCL2.d7/pre-b	IFNG.IL5.d28	-0.204	0.174	46
IL15.d7/pre-b	IFNG.IL5.d28	-0.204	0.174	46
OSM.d14/pre-b	IFNG.IL5.d28	0.202	0.174	47
CXCL12.d14/pre-b	IFNG.IL5.d28	0.201	0.175	47
IL17C.d14/pre-b	IFNG.IL5.d28	0.198	0.182	47
IL2.d1/pre-b	IFNG.IL5.d28	0.218	0.183	39
CSF1.d7/pre-b	IFNG.IL5.d28	-0.197	0.189	46
IL18.d14/pre-b	IFNG.IL5.d28	0.193	0.193	47
IL4.d7/pre-b	IFNG.IL5.d28	-0.194	0.197	46
CCL2.d14/pre-b	IFNG.IL5.d28	0.191	0.198	47
CXCL10.d1/pre-b	IFNG.IL5.d28	0.194	0.201	45
CCL7.d1/pre-b	IFNG.IL5.d28	-0.188	0.210	46
CCL7.d3/pre-b	IFNG.IL5.d28	-0.182	0.220	47
CXCL10.d3/pre-b	IFNG.IL5.d28	0.181	0.225	47
IL27.d1/pre-b	IFNG.IL5.d28	0.180	0.232	46
CCL4.d7/pre-b	IFNG.IL5.d28	0.179	0.235	46
LTA.d1/pre-b	IFNG.IL5.d28	0.176	0.242	46
CCL8.d14/pre-b	IFNG.IL5.d28	0.173	0.244	47
CCL2.d3/pre-b	IFNG.IL5.d28	-0.170	0.253	47
CCL4.d1/pre-b	IFNG.IL5.d28	0.172	0.254	46
VEGFA.d7/pre-b	IFNG.IL5.d28	-0.172	0.254	46
IL10.d14/pre-b	IFNG.IL5.d28	0.168	0.258	47
EGF.d3/pre-b	IFNG.IL5.d28	0.167	0.262	47
CSF1.d14/pre-b	IFNG.IL5.d28	0.166	0.264	47
CCL11.d1/pre-b	IFNG.IL5.d28	-0.165	0.274	46

TSLP.d7/pre-b	IFNG.IL5.d28	0.178	0.280	39
IL1B.d14/pre-b	IFNG.IL5.d28	0.160	0.281	47
LTA.d14/pre-b	IFNG.IL5.d28	0.159	0.285	47
IL17F.d14/pre-b	IFNG.IL5.d28	0.157	0.298	46
CCL7.d7/pre-b	IFNG.IL5.d28	-0.155	0.305	46
CSF2.d1/pre-b	IFNG.IL5.d28	-0.153	0.310	46
CCL19.d3/pre-b	IFNG.IL5.d28	0.148	0.320	47
TNFSF10.d1/pre-b	IFNG.IL5.d28	0.144	0.341	46
IL17A.d7/pre-b	IFNG.IL5.d28	0.142	0.348	46
MMP1.d1/pre-b	IFNG.IL5.d28	-0.141	0.349	46
IL10.d3/pre-b	IFNG.IL5.d28	-0.139	0.350	47
IFNG.d7/pre-b	IFNG.IL5.d28	-0.135	0.372	46
IL4.d1/pre-b	IFNG.IL5.d28	-0.135	0.372	46
CCL13.d7/pre-b	IFNG.IL5.d28	-0.134	0.375	46
IL7.d3/pre-b	IFNG.IL5.d28	0.132	0.376	47
CSF2.d3/pre-b	IFNG.IL5.d28	-0.132	0.377	47
IL33.d14/pre-b	IFNG.IL5.d28	0.131	0.380	47
IL13.d7/pre-b	IFNG.IL5.d28	-0.134	0.387	44
CCL13.d3/pre-b	IFNG.IL5.d28	0.128	0.392	47
CXCL11.d1/pre-b	IFNG.IL5.d28	0.131	0.393	45
OSM.d1/pre-b	IFNG.IL5.d28	-0.128	0.398	46
OLR1.d3/pre-b	IFNG.IL5.d28	0.126	0.399	47
TNF.d3/pre-b	IFNG.IL5.d28	0.125	0.401	47
IL2.d7/pre-b	IFNG.IL5.d28	0.140	0.402	38
IL7.d7/pre-b	IFNG.IL5.d28	-0.126	0.405	46
IL4.d3/pre-b	IFNG.IL5.d28	0.124	0.405	47
IL17A.d14/pre-b	IFNG.IL5.d28	0.121	0.417	47
IL33.d3/pre-b	IFNG.IL5.d28	0.120	0.421	47
CCL13.d14/pre-b	IFNG.IL5.d28	0.120	0.423	47
CCL8.d3/pre-b	IFNG.IL5.d28	0.119	0.424	47

IL7.d1/pre-b	IFNG.IL5.d28	0.121	0.424	46
CXCL12.d7/pre-b	IFNG.IL5.d28	0.120	0.425	46
IL10.d1/pre-b	IFNG.IL5.d28	-0.117	0.437	46
IL33.d1/pre-b	IFNG.IL5.d28	0.118	0.439	45
CCL8.d1/pre-b	IFNG.IL5.d28	0.117	0.440	46
MMP1.d3/pre-b	IFNG.IL5.d28	-0.115	0.440	47
CSF3.d1/pre-b	IFNG.IL5.d28	-0.116	0.441	46
OSM.d3/pre-b	IFNG.IL5.d28	0.115	0.442	47
TNFSF10.d14/pre-b	IFNG.IL5.d28	0.115	0.443	47
IL1B.d1/pre-b	IFNG.IL5.d28	0.114	0.451	46
CCL7.d14/pre-b	IFNG.IL5.d28	0.109	0.465	47
IL27.d7/pre-b	IFNG.IL5.d28	-0.109	0.470	46
CCL8.d7/pre-b	IFNG.IL5.d28	-0.108	0.474	46
IL33.d7/pre-b	IFNG.IL5.d28	-0.108	0.474	46
OLR1.d7/pre-b	IFNG.IL5.d28	0.105	0.486	46
CCL3.d1/pre-b	IFNG.IL5.d28	0.105	0.488	46
IL13.d3/pre-b	IFNG.IL5.d28	-0.104	0.503	44
IL2.d14/pre-b	IFNG.IL5.d28	0.111	0.506	38
CXCL8.d3/pre-b	IFNG.IL5.d28	0.099	0.510	47
OSM.d7/pre-b	IFNG.IL5.d28	0.100	0.510	46
IL7.d14/pre-b	IFNG.IL5.d28	0.097	0.518	47
FLT3LG.d3/pre-b	IFNG.IL5.d28	-0.088	0.555	47
IL13.d1/pre-b	IFNG.IL5.d28	0.093	0.557	42
CCL11.d3/pre-b	IFNG.IL5.d28	-0.085	0.570	47
IL17F.d7/pre-b	IFNG.IL5.d28	-0.085	0.577	45
CCL13.d1/pre-b	IFNG.IL5.d28	-0.084	0.579	46
IL6.d3/pre-b	IFNG.IL5.d28	-0.082	0.582	47
IL17A.d3/pre-b	IFNG.IL5.d28	0.082	0.583	47
CCL11.d14/pre-b	IFNG.IL5.d28	0.082	0.586	47
FLT3LG.d1/pre-b	IFNG.IL5.d28	-0.081	0.592	46

CXCL9.d7/pre-b	IFNG.IL5.d28	0.080	0.596	46
CSF2.d14/pre-b	IFNG.IL5.d28	0.079	0.596	47
IL17C.d7/pre-b	IFNG.IL5.d28	-0.079	0.601	46
CSF2.d7/pre-b	IFNG.IL5.d28	-0.078	0.606	46
MMP1.d14/pre-b	IFNG.IL5.d28	0.077	0.608	47
CXCL11.d7/pre-b	IFNG.IL5.d28	-0.076	0.615	46
LTA.d3/pre-b	IFNG.IL5.d28	0.075	0.618	47
EGF.d1/pre-b	IFNG.IL5.d28	0.074	0.627	46
TNFSF12.d7/pre-b	IFNG.IL5.d28	-0.072	0.635	46
CSF3.d14/pre-b	IFNG.IL5.d28	0.069	0.645	47
MMP1.d7/pre-b	IFNG.IL5.d28	-0.070	0.646	46
TNFSF10.d3/pre-b	IFNG.IL5.d28	-0.065	0.662	47
OLR1.d1/pre-b	IFNG.IL5.d28	-0.066	0.662	46
MMP12.d1/pre-b	IFNG.IL5.d28	-0.061	0.685	46
IL1B.d7/pre-b	IFNG.IL5.d28	-0.060	0.693	46
IL15.d3/pre-b	IFNG.IL5.d28	-0.058	0.699	47
TGFA.d1/pre-b	IFNG.IL5.d28	-0.058	0.700	46
MMP12.d3/pre-b	IFNG.IL5.d28	-0.057	0.702	47
TSLP.d14/pre-b	IFNG.IL5.d28	0.063	0.703	39
IL6.d1/pre-b	IFNG.IL5.d28	0.054	0.724	46
CSF3.d7/pre-b	IFNG.IL5.d28	0.050	0.740	46
IL18.d1/pre-b	IFNG.IL5.d28	0.049	0.746	46
IL17F.d1/pre-b	IFNG.IL5.d28	-0.049	0.748	45
CXCL12.d1/pre-b	IFNG.IL5.d28	0.047	0.759	46
IL15.d1/pre-b	IFNG.IL5.d28	0.045	0.765	46
LTA.d7/pre-b	IFNG.IL5.d28	-0.045	0.767	46
IL17A.d1/pre-b	IFNG.IL5.d28	0.044	0.770	46
CXCL12.d3/pre-b	IFNG.IL5.d28	0.044	0.770	47
CXCL10.d7/pre-b	IFNG.IL5.d28	0.043	0.775	46
IL10.d7/pre-b	IFNG.IL5.d28	0.041	0.785	46

HGF.d1/pre-b	IFNG.IL5.d28	-0.041	0.787	46
VEGFA.d1/pre-b	IFNG.IL5.d28	0.041	0.789	46
TGFA.d7/pre-b	IFNG.IL5.d28	0.040	0.790	46
IL4.d14/pre-b	IFNG.IL5.d28	-0.039	0.797	47
CXCL8.d7/pre-b	IFNG.IL5.d28	0.038	0.800	46
EGF.d7/pre-b	IFNG.IL5.d28	0.038	0.804	46
IL1B.d3/pre-b	IFNG.IL5.d28	0.037	0.806	47
CCL2.d1/pre-b	IFNG.IL5.d28	0.037	0.809	46
TNF.d7/pre-b	IFNG.IL5.d28	0.032	0.835	46
CSF1.d3/pre-b	IFNG.IL5.d28	-0.030	0.840	47
IL18.d7/pre-b	IFNG.IL5.d28	-0.029	0.850	46
CCL3.d7/pre-b	IFNG.IL5.d28	-0.027	0.856	46
CCL19.d1/pre-b	IFNG.IL5.d28	-0.027	0.861	46
TNFSF12.d1/pre-b	IFNG.IL5.d28	0.024	0.872	46
TSLP.d1/pre-b	IFNG.IL5.d28	-0.027	0.874	38
CSF3.d3/pre-b	IFNG.IL5.d28	0.024	0.874	47
IL13.d14/pre-b	IFNG.IL5.d28	-0.022	0.888	45
IL6.d7/pre-b	IFNG.IL5.d28	-0.021	0.890	46
TNFSF12.d3/pre-b	IFNG.IL5.d28	-0.020	0.894	47
HGF.d7/pre-b	IFNG.IL5.d28	0.020	0.895	46
IL17F.d3/pre-b	IFNG.IL5.d28	0.019	0.900	45
CSF1.d1/pre-b	IFNG.IL5.d28	-0.016	0.917	46
VEGFA.d3/pre-b	IFNG.IL5.d28	0.015	0.920	47
MMP12.d7/pre-b	IFNG.IL5.d28	-0.015	0.922	46
IL27.d3/pre-b	IFNG.IL5.d28	0.014	0.927	47
IL17C.d3/pre-b	IFNG.IL5.d28	-0.010	0.948	47
FLT3LG.d7/pre-b	IFNG.IL5.d28	0.010	0.950	46
IL18.d3/pre-b	IFNG.IL5.d28	-0.009	0.950	47
CXCL8.d1/pre-b	IFNG.IL5.d28	0.009	0.954	46
TSLP.d3/pre-b	IFNG.IL5.d28	-0.007	0.966	39

CCL11.d7/pre-b	IFNG.IL5.d28	-0.005	0.973	46
CCL19.d7/pre-b	IFNG.IL5.d28	-0.002	0.989	46
HGF.d3/pre-b	IFNG.IL5.d28	-0.001	0.993	47

Table S4. Antibodies details.

Target	Conjugate	Host	Target	Clone	Catalog	Vendor	Application	Dilution
IgG	PE	Mouse	Human	JDC-10	9040-09	SouthernBiotech	Ab response	1/50
IgG1	PE	Mouse	Human	HP6001	9054-09	SouthernBiotech	Ab response	1/250
IgG2	PE	Mouse	Human	HP6025	9070-09	SouthernBiotech	Ab response	1/50
IgG3	PE	Mouse	Human	HP6050	9210-09	SouthernBiotech	Ab response	1/50
IgG4	PE	Mouse	Human	HP6025	9200-09	SouthernBiotech	Ab response	1/50
CD4	APC-eF780	Mouse	Human	RPA-T4	47-0049-42	LIFE TECH	AIM Assay	1/50
CD3	AF700	Mouse	Human	UCHT1	56-0038-42	eBioscience	AIM Assay	1/50
CD8	V500	Mouse	Human	RPA-T8	560774	BD	AIM Assay	1/100
CD14	V500	Mouse	Human	M5E2	561391	BD	AIM Assay	1/100
CD19	V500	Mouse	Human	HIB19	561121	BD	AIM Assay	1/100
CD45RA	eF450	Mouse	Human	HI100	48-0458-42	Invitrogen	AIM Assay	1/50
CCR7	PerCP-Cy5.5	Mouse	Human	G043H7	353200	Biolegend	AIM Assay	1/25
OX40	PE-Cy7	Mouse	Human	Ber-ACT35	350012	Biolegend	AIM Assay	1/50
CD137	APC	Mouse	Human	4B4-1	309810	Biolegend	AIM Assay	1/50
CD25	FITC	Mouse	Human	M-A251	555431	BD	AIM Assay	1/50
CD69	BV605	Mouse	Human	FN50	562989	BD	AIM Assay	1/50
PDL1	PE	Mouse	Human	29E.2A3	329706	Biolegend	AIM Assay	1/50
